# Insight into the Synthesis and Characterization of Organophosphorus-Based Bridged Triazine Compounds

**DOI:** 10.3390/molecules24142672

**Published:** 2019-07-23

**Authors:** Khalifah A. Salmeia, Antonia Neels, Dambarudhar Parida, Sandro Lehner, Daniel Rentsch, Sabyasachi Gaan

**Affiliations:** 1 Additives and Chemistry, Advanced Fibers, Empa Swiss Federal Laboratories for Materials Science and Technology, Lerchenfeldstrasse 5, 9014 St. Gallen, Switzerland; 2 Center for X-ray Analytics, Swiss Federal Laboratories for Materials Science and Technology, Empa, Überlandstrasse 129, 8600 Dübendorf, Switzerland; 3 Laboratory for Functional Polymers, Empa Swiss Federal Laboratories for Materials Science and Technology, Überlandstrasse 129, 8600 Dübendorf, Switzerland

**Keywords:** Michaelis–Arbuzov rearrangement, cyanuric chloride, triazine derivatives, organophosphorus compounds

## Abstract

In this article, we report the synthesis of 2,4,6-substituted *s*-triazine-based organophosphorus compounds via a two-step process, which enables their production in high yields, and with a high purity as solids. In the first step, a Michaelis–Arbuzov rearrangement of cyanuric chloride with triethyl phosphite afforded 2,4,6-trisdiethoxyphosphinyl-1,3,5-triazine (HEPT). Subsequently, the nucleophilic substitution reaction on the triazine carbon was achieved, owing to the electron-withdrawing ability of the phosphonate groups. This characteristic of HEPT facilitated its derivatization with bi-functional amines, producing novel P–C containing bridged triazine organophosphorus compounds. The molecular structures of all of the compounds were confirmed by NMR spectroscopy, CHN elemental analysis, and single crystal X-ray analysis. In the thermogravimetric analysis in an N_2_ environment, >33% char formation was observed for the bridged compounds. The chemical composition analysis of the char obtained under the oxidative thermal decomposition of the bridged compounds confirmed the presence of phosphorus- and nitrogen-enriched species, which indicate their function in the condensed phase. Comparatively, the detection of HPO and H–C≡P in the gas phase during the pyrolysis of the bridged compounds can act as a source for PO^•^, which is known for its gas phase flame inhibition reactions. The synergy of significant char formation and the generation of intermediates leading to PO^•^ during pyrolysis makes these molecules promising flame-retardant additives.

## 1. Introduction

**Scheme 1:**
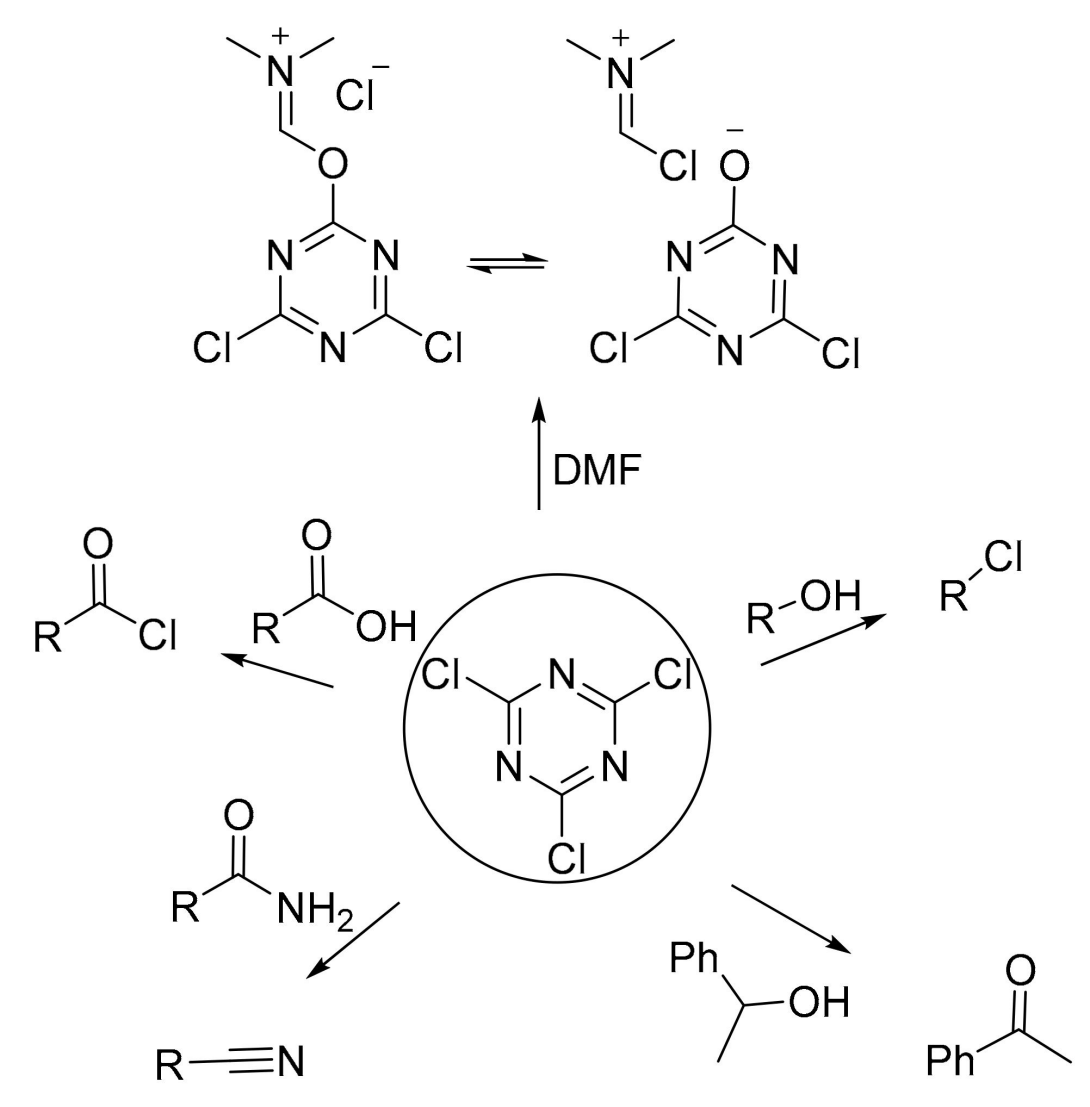
General schematic example for using cyanuric chloride as a chemical reagent.

Nitrogen-containing heterocycles (azaheterocycles) are common organic compounds that play a crucial role in different applications. Although the symmetric 1,3,5-triazine, which is referred to as s-triazine, is considered monotonous [1], new synthetic approaches were investigated to produce new substituted 1,3,5-triazine derivatives [2]. The most common chemical reaction hereof is the substitution of the chlorine atom of the commercially available 2,4,6-trichloro-1,3,5-triazine (cyanuric chloride) with nucleophiles [3]. Cyanuric chloride has been known since the early 1800, and has a similar reaction profile to acyl chlorides, rather than alkyl chlorides; since then, it has received great attention as a chemical reagent for different chemical reactions, as shown in Scheme 1 [4,5].

Moreover, the ability to replace the chlorine atom (Cl) in the presence of an acid scavenger was found to produce mono-, di-, and tri-substituted s-triazines [3]. The first displacement of a Cl atom happens between 0 °C to ambient temperature, and the subsequent replacement of Cl occurs at higher reaction temperatures [2,6]. The phosphorus-bearing azaheterocyclic compounds have received great attention because of their different applications in medicinal chemistry and agrochemicals [7]. Industrially, more than 70% of cyanuric chloride production is used for the production of pesticides and herbicides [8]. Substituted 1,3,5-triazine derivatives could find an application as flame retardant additives [9,10], and their flame retardant performance was investigated extensively on cotton [11,12,13,14,15], epoxy resins [16,17,18], and polypropylene [19,20,21,22].

The nitrogen-containing flame retardants work either in gas phase or in condensed phase. These flame-retardant derivatives, which release a large amount of non-flammable gases upon decomposition, act predominantly in the gas phase. On the other hand, the additives that lead to the formation of acidic derivatives, act in the condensed phase [23]. It is known that organophosphorus flame-retardant additives act predominantly in the gas phase, producing PO**^•^** species during thermal decomposition, which quench the radical species (H**^•^** and **^•^**OH), thereby inhibiting the cycle of fire [24,25]. Phosphorus-containing additives liberate phosphoric acid during their decomposition, and act synergistically together with nitrogen-containing additives in improving the char formation [26]. The synergism of P,N-containing flame retardants was extensively investigated, for enhancing both the gas- and condensed-phase mode of action [17,27]. Triazine-based flame-retardant additives can act in both the gas and condensed phases simultaneously [6,9]. Notwithstanding, the triazine compound itself was found to be a good char forming agent [12,17,19,21,26,28,29]. Despite the increase in the number of synthetic methodologies, popular methods for phosphonylation of cyanuric chloride include the Michaelis–Arbuzov rearrangement and Michaelis–Becker reaction. The Michaelis–Arbuzov rearrangement of cyanuric chloride with trialkyl phosphite was investigated to produce 2,4,6-trisdialkoxyphosphinyl-1,3,5-triazine [30]. This type of reaction can occur vigorously with neat trialkyl phosphite at ambient conditions, and can take place in boiling solvents. The partial substitution of the chlorine atom of cyanuric chloride via the Michaelis–Arbuzov rearrangement has also been investigated [31]. However, the ability of the nucleophilic substitution of the 2-chloro-atom of 4,6-substituted 1,3,5-triazine decreases with the lessening electron-withdrawing effect of its substituents. The electron-withdrawing substituents reduce the electron density on the carbon atoms of the triazine ring, making it more susceptible to a nucleophilic reaction. A new class of phosphino-derivatives of s-triazine was also synthesized using three different synthetic methodologies [32].

Herein, we report a two-step synthetic of P-containing bridged triazine compounds using cyanuric chloride as a starting material. This synthetic process enabled the preparation of bridged triazine compounds in high yields and with a high purity as solids. The synthesis of the ethylene diamine-based bridged triazine compound is already reported in the literature, however, its detailed chemical and thermal characterizations are missing [33]. Bridged compounds, being larger structures, are expected to be thermally more stable. Additionally, the bridged characteristic of the triazine structures could decrease their chemical reactivity, which is an important requirement for the high-temperature processing of polymers. Moreover, the addition of a nitrogen bridge in the structure alters the P:N ratio, which has a strong influence on their flame retardant behavior [34]. The chemical structures of these compounds were characterized by NMR spectroscopy, elemental analysis, and single crystal X-ray analysis. Furthermore, we have performed their detailed thermal analysis using thermogravimetric analysis (TGA) and direct insertion probe mass spectrometry (DIP-MS).

## 2. Results and Discussion

### 2.1. Synthesis of Chemicals

**Scheme 2:**
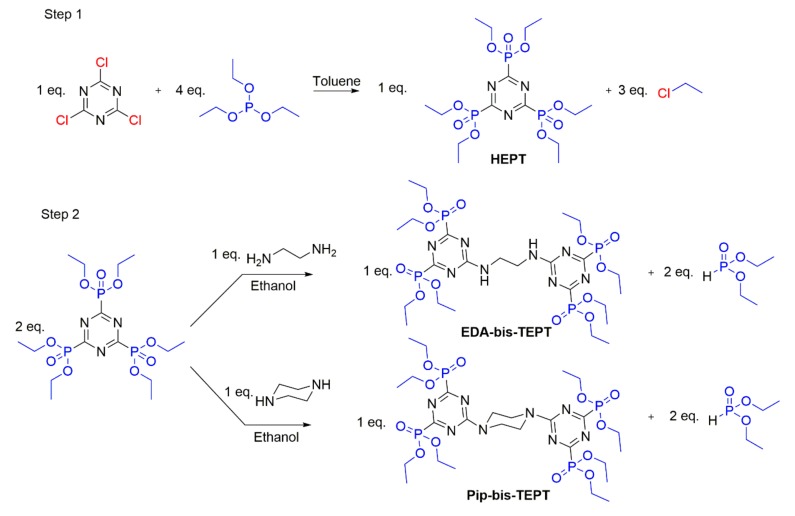
The synthesis scheme of triazine bridged derivatives of 2,4,6-trisdiethoxyphosphinyl-1,3,5-triazine (HEPT) and the final products *N*,*N*′-Bis[4,6-bis(diethylphosphono)-1,3,5-Triazin-yl]-1,2-diaminoethane (EDA-bis-TEPT) and *N*,*N*′-Bis[4,6-bis(diethylphosphono)-1,3,5-Triazin-yl]-piperazine (Pip-bis-TEPT).

The synthetic methodology to obtain the bridged triazine derivatives is shown in Scheme 2. The Michaelis–Arbuzov rearrangement of cyanuric chloride with triethyl phosphite afforded 2,4,6-trisdiethoxyphosphinyl-1,3,5-triazine (HEPT; Scheme 2, step 1). It was then used as an intermediate to synthesize *N*,*N*′-Bis[4,6-bis(diethylphosphono)-1,3,5-Triazin-yl]-1,2-diaminoethane (EDA-bis-TEPT) and *N*,*N*′-Bis[4,6-bis(diethylphosphono)-1,3,5-Triazin-yl]-piperazine (Pip-bis-TEPT) (Scheme 2, step 2).

It is noteworthy to mention that only three equivalents of triethyl phosphite, with respect to cyanuric chloride, are enough to produce the precursor HEPT. However, in order to obtain a high product yield, four equivalents of HEPT were used. Attempts to partially replace the three Cl-atoms via the Michaelis–Arbuzov rearrangement to prepare di-substituted triazine derivatives in a high yield and with a high purity were unsuccessful. This may be attributed to the electron-withdrawing effect of the phosphonate group, which reduces the electron density of the triazine ring and enhances the activity of the Cl-atom for further substitution reactions, such as preferring the tri-substituted product [31]. The substitution of one phosphonate group of HEPT with the amine group, to produce EDA-bis-TEPT and Pip-bis-TEPT, was performed following a previously reported procedure [10,30,35]. It is reported that the substitution of the phosphonate group is affected by the reaction solvent, using ammonia as a nucleophile [35]. By using ethanol as a reaction solvent, two phosphonate groups were replaced by the amine group, whilst one group was replaced when the reaction was performed in benzene. In the case of this work, the substitution reaction was carried out in ethanol at ambient conditions, to which only one phosphonate group of HEPT was selectively replaced with an amine group in a high yield, and no by-products were detected. It can be assumed that by replacing one phosphonate group with an electron-donating atom, such as nitrogen, the electron density of the triazine ring increases, thus decreasing the reactivity of the ring for further substitution reactions at ambient conditions [31]. On the contrary to the previous report [33], this methodology led to producing pure products that were isolated as solids. Hence, the correlation between the NMR spectroscopy and single crystal X-ray analysis provided an insight into the chemical structure of these products.

### 2.2. Characterization of the Molecular Structure of Chemicals

**Figure 1:**
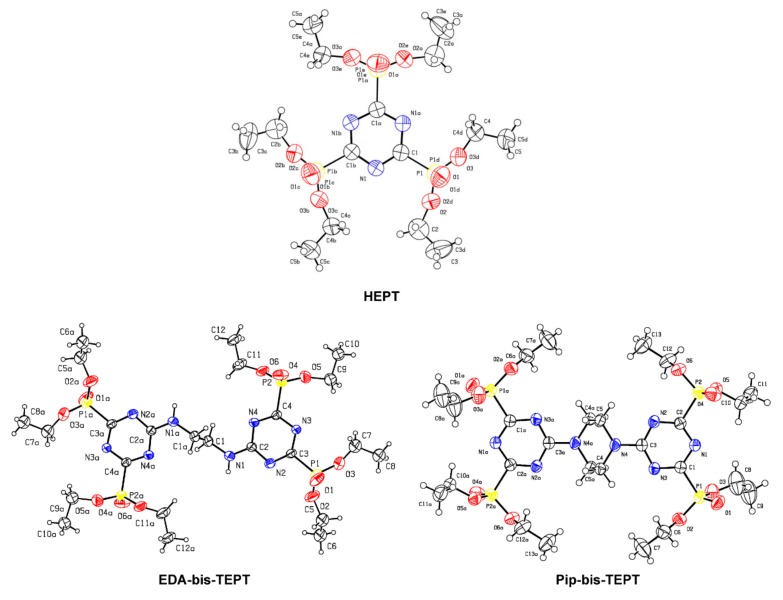
Molecular structures of HEPT, EDA-bis-TEPT, and Pip-bis-TEPT. Thermal ellipsoids are drawn with a 50% probability level [37]. Phosphorus (yellow), oxygen (red), and nitrogen (blue).

All of the synthesized chemicals were analyzed by 1D and 2D ^1^H, ^13^C, and ^31^P-NMR spectroscopy. The ^31^P-NMR data of the target materials showed high-frequency shifts of the ^31^P-NMR resonances as soon as one of the phosphonate groups of the precursor HEPT was replaced by an amine group, producing EDA-bis-TEPT and Pip-bis-TEPT (Figures S1, S2A, and S3A). From the chemical structures, all of the phosphorus atoms were expected to have the same chemical environment, and the presence of one signal for each compound in their corresponding ^13^P-NMR spectra was expected. However, the ^31^P-NMR spectrum of EDA-bis-TEPT showed two resonances assigned to an AB spin system, with a ^31^P–^31^P coupling constant of about 8.8 Hz. Owing to the presence of the electron-donating NH groups next to the triazine ring, the two phosphorous atoms became inequivalent, and consequently, two different signals were observable at ambient temperatures [33]. The electron-donating ability of the NH group could influence the electron densities around the triazine carbons of EDA-bis-TEPT that bind directly to the phosphorus atoms, as confirmed by X-ray analysis (Table 1 and Figure 1). Hence, the electron densities around these carbon atoms are inequivalent, and one carbon atom has more electron density than the other carbon atom. Accordingly, the ^13^C-NMR spectrum of EDA-bis-TEPT showed two doublet of doublets for the carbon atoms that directly bind to the phosphorus atoms in the same ring (Figure S2A). In contrast, each phosphonate group on one ring was found to be identical to the other group on the other triazine ring. The ^13^C-NMR analysis showed a low-frequency chemical shift of the triazine carbon when a phosphonate group was replaced by a nitrogen atom, from 171.2 ppm observed for HEPT**,** to 163.2 ppm and 161.8 ppm for EDA-bis-TEPT and Pip-bis-TEPT, respectively (Figures S1, S2A, and S3A). This can be attributed to the increase of electron density on the triazine carbon atom that binds to the amine group, because of the electron-donating ability of the nitrogen atom.

The solid-state chemical structures of all of the compounds were confirmed by the single crystal X-ray analysis (Figure 1). HEPT crystallizes in a centrosymmetric hexagonal cell, in space group P6_3_/m. The molecular formula corresponding to the crystallographic asymmetric unit is (C_15_H_30_N_3_O_9_P_3_). The molecule is on a mirror plane, resulting in a positional disorder with 50% atomic position occupation of all P and O atoms; the C1 and N1 atoms are precisely positioned on the mirror plane, and the remaining C atoms are also 50% occupied, related to the positional disorder. EDA-bis-TEPT crystallizes in a centrosymmetric triclinic cell, in space group P-1. The molecular formula corresponds to (C_24_H_46_N_8_O_12_P_4_), and all of the molecules lie on symmetry centers. The crystallographic asymmetric unit is composed out of three half molecules. Ten out of twelve ethyl groups have been disordered. The positional disorder has been refined, with a 50% atomic position occupation of all of the concerned C and H atoms, and the thermal vibration has been constrained to be equal. The positional disorder encountered for this structure results in a weak diffraction power of the crystal. However, the crystal structure has been refined, with reasonable standard uncertainties on atomic positions, bond lengths, and angles. Pip-bis-TEPT crystallizes in a centrosymmetric triclinic cell, space group P-1. The molecular formula is (C_26_H_48_N_8_O_12_P_4_); the asymmetric unit is one half of the molecule, as the molecule is positioned on an inversion center. All of the structures were solved by direct methods using the program SHELXS [36], and refined by full-matrix least-squares on F^2^ with SHELXL [37]. The hydrogen atoms were included in the calculated positions, and were treated as riding atoms using SHELXL-97 default parameters. All of the non-hydrogen atoms were refined anisotropically, and no absorption correction was applied.

It was found that the P–C_triazine_ bond distance of HEPT (1.846 (4) Å) became shorter when one phosphonate group was replaced with the corresponding amine functional group, affording either EDA-bis-TEPT or Pip-bis-TEPT (Table 1).

It is noteworthy to mention that the P–C_triazine_ bond distance within either EDA-bis-TEPT or Pip-bis-TEPT are not relatively equal. For example, the bond distances of P2–C4 and P1–C3 in EDA-bis-TEPT (Figure 1) are 1.834 (6) and 1.827 (5) Å, respectively. The same was observed in Pip-bis-TEPT, as the bond distance of P1–C1 is 1.826 (3) Å, and for P2–C2 is 1.830 (3) Å. The decrease of the P–C_triazine_ bond distance in EDA-bis-TEPT and Pip-bis-TEPT in comparison to HEPT indicates that the bond of the phosphonate group to the triazine ring became stronger by replacing one phosphonate group with the amine group.

**Scheme 3:**
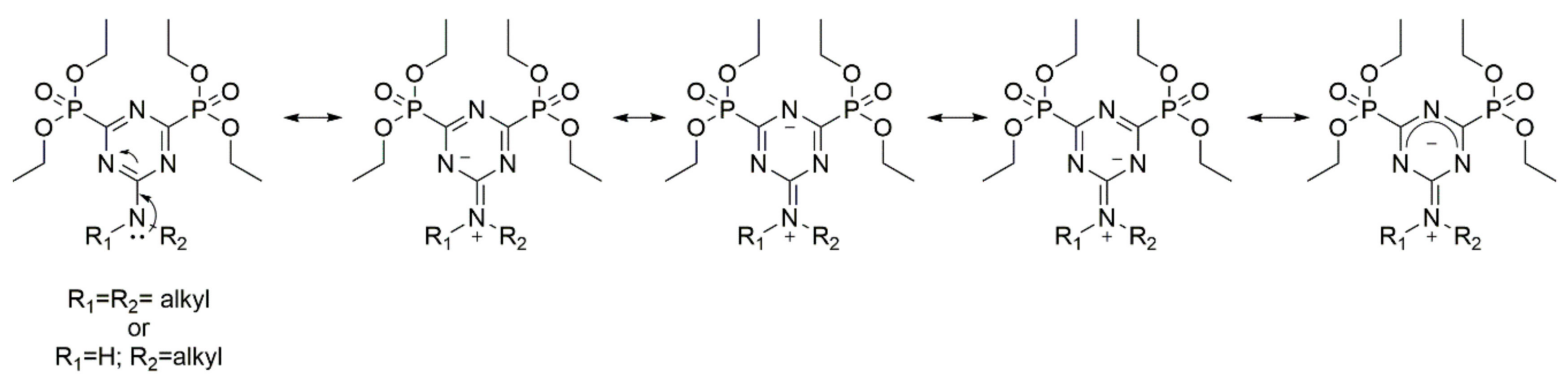
Representative scheme showing the electronic resonance in a triazine ring because of the electron-donating ability of the amine group.

The correlation of the NMR spectroscopy and single-crystal X-ray analysis shows that the electron density around the phosphonate groups decreased, which can be attributed to the electron-donating nature of nitrogen, which enhances the electron density of the triazine ring. Therefore, the electron-withdrawing ability of the phosphonate groups decreased, which may, in turn, reduce the eligibility of the triazine ring for further substitution reactions at ambient conditions [31]. This can be observed in the NMR spectra, because of the difference in the chemical shifts of the phosphorus and carbons atoms in EDA-bis-TEPT and Pip-bis-TEPT, in comparison to the precursor HEPT. The X-ray data show that the lengths of the C–N bonds in the triazine ring in compounds EDA-bis-TEPT and Pip-bis-TEPT were found to be non-equal with respect to the precursor, HEPT. For example, the bond distances of C–N of the triazine ring, which bind to the bridged amine groups, are relatively longer than those that are close to the phosphonate groups. The nature of the amine group as an electron-donor by resonance leads to the donation of its free-electron to the triazine ring, which increases the electron density of the triazine ring, but deteriorates the distribution of the electron density, as shown in Scheme 3.

### 2.3. Thermal Characteristics and Chemical Composition of the Resulting Chars

**Figure 2:**
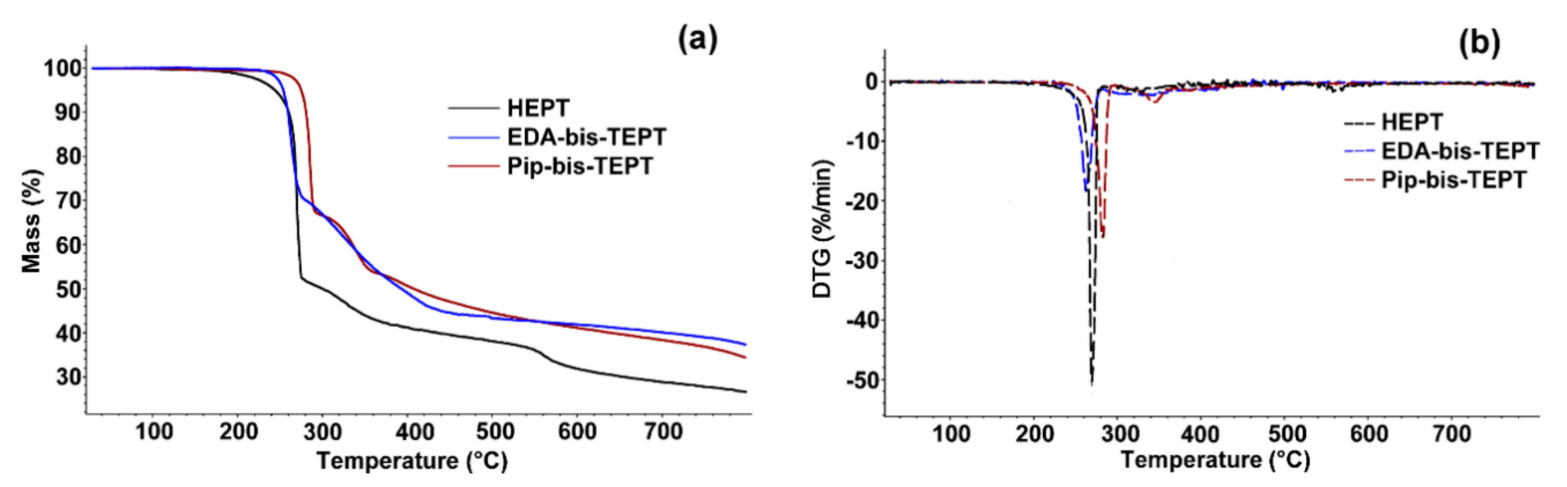
Thermal gravimetric analysis (TGA) (**a**), and derivative thermogravimetry (DTG) (**b**) plots of the organophosphorus-based bridged triazine derivatives under an N_2_ atmosphere.

The thermal gravimetric analysis (TGA) is an established technique that provides a quantitative physical and chemical phenomena, including the thermal decomposition and char formation in milligram sample quantities [24]. The thermal stability of HEPT, EDA-bis-TEPT, and Pip-bis-TEPT was measured in TGA (Figure 2), and the data are summarized in Table 2.

**Figure 3:**
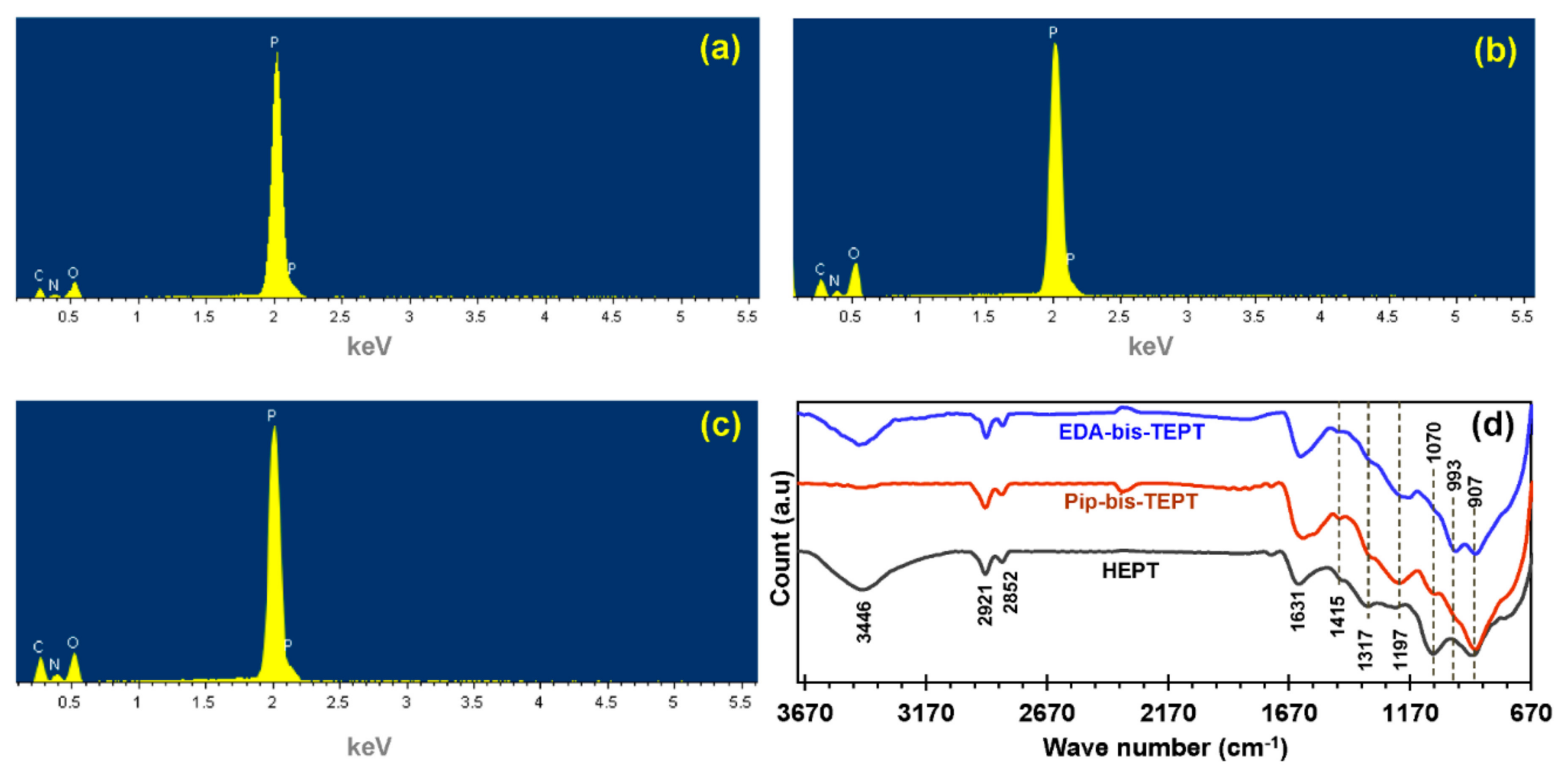
Energy-dispersive X-ray analysis (EDX) elemental composition of the char residue of HEPT (**a**), EDA-bis-TEPT (**b**), Pip-bis-TEPT (**c**), and the Fourier-Transform Infrared Spectroscopy (FTIR) spectra for their corresponding char residues (**d**). All of the char residues were obtained after the oxidative pyrolysis of the corresponding compounds.

As shown in Figure 2 and Table 2, all three of the compounds exhibited a significant residual char upon their pyrolysis under the N_2_ atmosphere. It is noticed that, by increasing the percentage ratio of nitrogen to phosphorus atoms in the corresponding molecules, the char residue increases. The elemental composition of residue after the oxidative decomposition of the samples, imitating the oxidative decomposition that can happen upon combustion, was investigated using energy-dispersive X-ray analysis (EDX; Figure 3a–c). All of the data are summarized in Table 3.

As shown in Table 3, the char residues of each compound retained about 40% of the original phosphorus content, with respect to their corresponding compounds. The retained phosphorus could play a crucial role in the condensed phase, and could contribute towards the char formation. The loss in phosphorus content can be attributed to the formation of PO^•^ species in the gas phase. Identical results were also observed from the percentage mass loss of nitrogen content and the retained amount in the residual char. On the other hand, the percentage mass loss of carbon content was more than 80%. This can be attributed to the accelerated formation of CO_2_ and H_2_O, upon the oxidative pyrolysis of the hydrocarbon contents of the molecules. The retained amount of carbon was enough for the formation of voluminous char.

The chemical composition of the residual chars obtained by the oxidative pyrolysis of all of the compounds were qualitatively studied by Fourier-Transform Infrared Spectroscopy (FTIR) analysis (Figure 3d). The bands at 907 cm^−1^ and 993 cm^−1^ were attributed to the existence of P–O–P and P–O–C bonds, respectively [19]. A high intensity of 907 cm^−1^ and 993 cm^−1^ suggests that the main composition of char is pyrophosphate and polyphosphates, which is consistent with the EDX analysis. The band around 1317 cm^−1^ can be attributed to the P=O stretch [38], and at 1415 cm^−1^ it is because of the presence of P–N structures in the char [39]. Evidence of a carbonization reaction (C=C formation), which promotes char formation, can be seen as the presence of a strong band at 1631 cm^−1^ [39]. The bands at 2852 cm^−1^ and 2921 cm^−1^ were corresponding to the stretching vibration of the C–H bond, and the broad band at 3446 cm^−1^ could be for the stretching vibration of the N–H bond [40]. Surprisingly, the IR spectrum of the Pip-bis-TEPT char showed a relatively low intensity for the N–H bond in comparison to other char residues.

### 2.4. Direct Insertion Probe Mass Spectrometry (DIP-MS) Measurements

**Figure 4:**
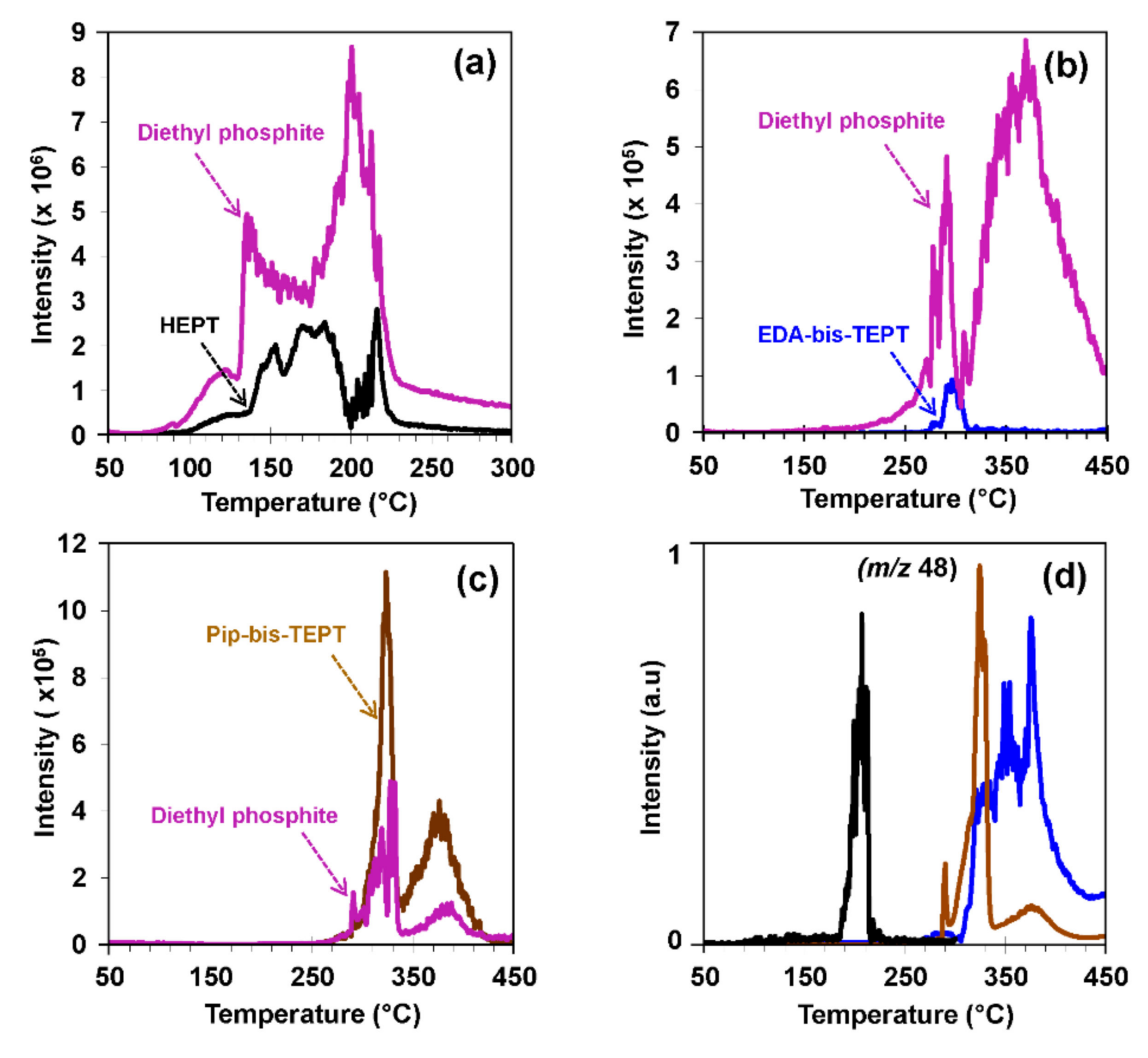
Extracted ion chromatogram of the synthesized compounds in comparison to their release of diethyl phosphite (**a**–**c**) and production of HPO (*m/z* 48) (**d**).

The above-mentioned SEM-EDX and FTIR study on residual char shed light on the possible mode of action of HEPT, EDA-bis-TEPT, and Pip-bis-TEPT in the condensed phase. However, about 75% to 80% of the products were lost in the gas phase. To identify the volatile products that evolved during their pyrolysis, DIP-MS measurements were performed, and the total ion chromatogram for the decomposition was investigated as a function of the temperature. The total ion chromatogram of each product revealed the release of diethyl phosphite (*m/z* 138) for all of the compounds, in addition to the volatilization of the main compounds, namely HEPT (*m/z* 489), EDA-bis-TEPT (*m/z* 763), and Pip-bis-TEPT (*m/z* 789), as illustrated in Figure 4a–c. The change of intensities between each product and its corresponding diethyl phosphite illustrate its gas phase mode of action. For example, comparing the intensities of all of the products with their corresponding release of diethyl phosphite (Figure 4a–c) can clarify their eligibility to act in the gas phase as flame retardant additives. A comparison of HEPT (Figure 4a) and EDA-bis-TEPT (Figure 4b) with their corresponding diethyl phosphite shows a relatively high intensity of the latter compound. That is to say, both compounds can have efficacy in the gas phase. The low intensity of EDA-bis-TEPT regarding diethyl phosphite (Figure 4b) indicates that its pyrolysis can happen faster than its evaporation. On the contrary to HEPT and EDA-bis-TEPT, Pip-bis-TEPT showed more thermal stability, as its evaporation is in concurrent with its pyrolysis.

Diethyl phosphite is considered an efficient flame retardant that acts in the gas phase, with the potential to produce PO^•^. Despite the difficulty of tracking this species using DIP-MS, a minor product was detected for all of the compounds at *m/z* 48, which can be attributed to HPO (Figure 4d), which upon its pyrolysis, may produce PO^•^. Moreover, another minor peak was detected for all of the compounds at *m/z* 44, which can be attributed to the production of CO_2_. However, as all of the measurements in DIP-MS were performed in an inert atmosphere, which may exclude the production of CO_2_, the production of H–C≡P instead of CO_2_ can be expected [41].

## 3. Materials and Methods

### 3.1. Materials

Cyanuric chloride (99% purity), triethyl phosphite (98% purity), ethylenediamine (*ReagentPlus*^®^, ≥99% purity), and Piperazine (*ReagentPlus*^®^, ≥99% purity) were purchased from Sigma-Aldrich (Buchs, Switzerland). Toluene (HPLC grade) was purchased from Biosolve BV (Valkenswaard, Netherlands), and absolute ethanol was purchased from Honeywell Fluka (Reinach, Switzerland). All of the chemicals and solvents were used as received, without any further purification.

### 3.2. Thermal Characterization, DIP-MS, SEM-EDX, Elemental Analysis, and NMR Spectrometer

The thermogravimetric analysis (TGA) was performed on a NETZSCH TG 209 F1 instrument (NETZSCH-Gerätebau GmbH, Selb, Germany). Tests were conducted on 2–5 mg samples at a heating rate of 10 °C/min, from 25 °C to 800 °C. The TGA curves represent the average of two measurements. Differential scanning calorimetry (DSC) analyses were performed on the DSC 214 Polyma instrument (NETZSCH-Gerätebau GmbH, Selb, Germany). All of the experiments were conducted at a controlled heating rate of 10 °C/min, under a nitrogen flow of 50 mL/min. A direct insertion probe mass spectrometry (DIP-MS) analysis was conducted for a 1–2 µg sample, using ThermoQuest FINNIGAN apparatus (Austin, TX, USA). The probe was heated from 30 °C to 300 °C for HEPT, and from 30 °C to 450 °C for EDA-bis-TEPT and Pip-bis-TEPT, at a rate of 10 K/min and 10^−6^ mbar pressure. Energy-dispersive X-ray spectroscopy (EDX) was applied in combination with a scanning electron microscope (SEM), using an Inca X-sight device from Oxford Instruments (EDX) and a S-4800 microscope from Hitachi (SEM) (Tokyo, Japan). For each sample, the EDX analysis was performed at two different areas on completely charred samples obtained from TGA, and an average value was obtained from the resulting elemental compositions. An estimated area of 100 μm × 100 μm was scanned for the EDX measurements using an acceleration voltage of the electron beam of 20 kV, an emission current of 15μA, and a working distance of 15mm. Under these experimental conditions, the information depth is in the order of a couple of microns. The micro-elemental analysis (CHN) was performed by the molecular and biomolecular analysis service (MoBiAS) of the Laboratory of Organic Chemistry at Swiss Federal Institute of Technology in Zurich (ETHZ). The ^1^H, ^13^C, and ^31^P-NMR data were recorded on a Bruker Avance III 400 NMR spectrometer (Bruker Biospin AG, Fällanden, Switzerland) at 400.2, 100.6, and 162.0 MHz, respectively. The 1D and the 2D ^1^H–^13^C HSQC and ^1^H–^13^C HMBC correlation NMR experiments used for the full resonance assignments were performed on a 5 mm CryoProbe™ Prodigy probe equipped with a z-gradient at 298 K, using the Bruker standard pulse programs and parameter sets, applying 90° pulse lengths of 11.4 μs (^1^H), 10.0 μs (^13^C), and 12.0 μs (^31^P). The ^1^H and ^13^C chemical shifts (δ) in ppm are calibrated to residual solvent peaks (DMSO-*d*_6_: δ = 2.49 and 39.5 ppm), the ^31^P chemical shifts were referenced to an external sample with neat H_3_PO_4_ at 0.0 ppm. Wherever possible, the ^1^H–^31^P and ^31^P–^13^C coupling constants are reported in Hz. For ^13^C-NMR data multiplicities, *s* = quaternary carbon, *d* = CH, *t* = CH_2_, and *q* = CH_3_ are shown.

### 3.3. Single Crystal X-Ray Structure Determination

The crystals of HEPT, EDA-bis-TEPT, and Pip-bis-TEPT were grown by slow evaporation of their corresponding ethanol solution. Suitable crystals were selected and mounted on a Stoe Mark II-Imaging Plate Diffractometer System (Stoe and Cie, 2015), equipped with a graphite-monochromator (Darmstadt, Germany). Data collection was performed at –100 °C using Mo-Kα radiation (λ = 0.71073 Å). There were 180 exposures (5 min per exposure) that were obtained, at an image plate distance of 135 mm, φ = 0° and 0 < ω < 180°, with the crystal oscillating through 1° in ω. The resolution was D_min_–D_max_ 24.00–0.82 Å. The structures were solved by direct methods using the program SHELXS (Sheldrik, Göttingen, Germany) [36], and refined by full-matrix least-squares on F^2^ with SHELXL (Sheldrik, Göttingen, Germany) [37]. The hydrogen atoms were included in the calculated positions, and treated as riding atoms using SHELXL-97 default parameters. All of the non-hydrogen atoms were refined anisotropically. No absorption correction was applied. More refinement details for HEPT, EDA-bis-TEPT, and Pip-bis-TEPT are given in the supporting information. CCDC 1893600-1893602 contain the supplementary crystallographic data for compounds HEPT, EDA-bis-TEPT and Pip-bis-TEPT, respectively. These data can be obtained free of charge from The Cambridge Crystallographic Data Centre, via www.ccdc.cam.ac.uk/data_request/cif. The crystal data and refinements are summarized in Table 4.

### 3.4. Synthesis

#### 3.4.1. Synthesis of 2,4,6-Trisdiethoxyphosphinyl-1,3,5-Triazine (HEPT)

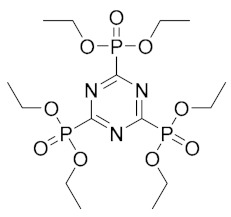



A five-neck double-jacket reactor (5 L) was equipped with a heating system, mechanical stirrer, reflux condenser, dropping funnel, and connected to a cooling trap. The reactor was purged with N_2_, then charged with cyanuric chloride (268.9 g; 1.46 mol) and anhydrous toluene (2.5 L). The solution was then heated to 60 °C, and triethyl phosphite (969.0 g; 5.83 mol) was added dropwise in a rate that the reaction temperature did not exceed 80 °C. After the complete addition, the reaction solution was stirred at 70 °C for 4 h, and then allowed to cool down to ambient temperature. The volatiles were then completely removed in a vacuum, affording a pale-yellow solid that was washed thoroughly with heptane. The solid was then collected by filtration, and dried overnight in a vacuum oven at 60 °C, affording white crystals. The yield was as follows: 691.4 g (97%); m.p. 96 °C. ^1^H-NMR (400.2 MHz, DMSO-*d*_6_) δ (ppm) was as follows: 4.28 (qt, *J*_H,H_ = 7.1 Hz, *J*_H,P_ = 7.3 Hz, 12H, O–C**H**_2_–CH_3_); 1.32 (t, *J*_H,H_ = 7.1 Hz, 18H, O-CH_2_-C**H**_3_). ^13^C-NMR (100.6 MHz, DMSO-*d*_6_) δ (ppm) was as follows: 171.2 (sdt, *J*_C,P_ = 257, 12.5 Hz, Cq); 64.4 (t ^a)^, O-**C**H_2_–CH_3_); 16.14 (q ^a)^, O-CH_2_–**C**H_3_). ^31^P-NMR (162.0 MHz, DMSO-d_6_) δ (ppm) was as follows: 1.4.

^a)^ Signals exhibit higher-order multiplet splitting because of *J*(P,C).

#### 3.4.2. Synthesis of *N*,*N*′-Bis[4,6-Bis(diethylphosphono)-1,3,5-Triazin-yl]-1,2-Diaminoethane (EDA-bis-TEPT)

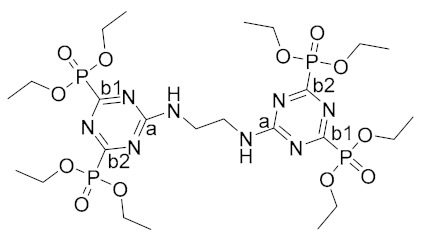



A two-neck round-bottomed flask (500 mL) was charged with HEPT (30.0 g; 61.3 mmol) in absolute ethanol (100 mL). Ethylenediamine (1.82 g; 30.4 mmol) was then dropwise added at ambient temperature. After the complete addition, the yellow solution was allowed to stir at ambient temperature. After 4 h, the volatiles were completely removed in a vacuum, affording a viscous yellow oil. Toluene (200 mL) was then added with vigorous stirring, affording a white precipitate. The solid was then collected by filtration, and dried overnight in a vacuum oven at 80 °C. The yield was as follows: 19.0 g (81%); m.p. 150 °C. ^1^H-NMR (400.2 MHz, DMSO-*d*_6_) δ (ppm) was as follows: 8.99 (m, 2H, N**H**); 4.21 (m, 16H, O–C**H**_2_–CH_3_); 3.56 (m, 4H, N–C**H**_2_–C**H**_2_–N); 1.28 (m, 24H, O–CH_2_–C**H**_3_). ^13^C-NMR (100.6 MHz, DMSO-*d*_6_) δ (ppm) was as follows: 170.9 (sdd ^a)^, *J*_C,P_ = 260, 14.6 Hz, 2C, C-b1); 170.2 (sdd ^a)^, *J*_C,P_ = 261, 15.2 Hz, 2C, C-b2); 163.2 (st, *J*_C,P_ = 18.1 Hz, 2C, C-a); 63.7 (tdd, *J*_C,P_ = 22.6, 6.1 Hz, 8C, O–**C**H_2_–CH_3_); 39.1 (t, 2C, N–**C**H_2_–**C**H_2_–N); 16.1(tdd, *J*_C,P_ = 5.8, 2.0 Hz, 8C, O–CH_2_–**C**H_3_). ^31^P-NMR (162.0 MHz, DMSO-*d*_6_) δ (ppm): 3.1 (d ^a)^, *J*_P,P_ = 8.8, P-b1); 2.9 (d ^a)^, *J*_P,P_ = 8.8, P-b2)).^1^H–^13^C HMBC: N**H** → N–**C**H_2_–**C**H_2_–N, **C**-a; O–C**H**_2_CH_3_ → O–CH_2_–**C**H_3_; N–C**H**_2_–C**H**_2_–N → N–**C**H_2_–**C**H_2_–N, **C**-a; O–CH_2_C**H**_3_ → O–**C**H_2_–CH_3_. The following analysis was calculated for C_24_H_46_N_8_O_12_P_4_: C, 37.80; H, 6.08; N, 14.69. The following was found: C, 37.60; H, 5.99; N, 14.57.

^a)^ Because the NH group positions “b1” and “b2” are not chemically equivalent, resonance assignments to b1 or b2 arbitrarily.

#### 3.4.3. Synthesis of *N*,*N*′-Bis[4,6-Bis(diethylphosphono)-1,3,5-Triazin-yl]-Piperazine (Pip-bis-TEPT)

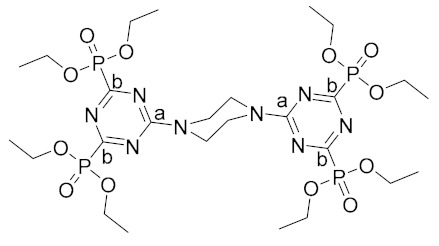



A two-neck round-bottomed flask (500 mL) was charged with HEPT (30.0 g; 61.3 mmol) in absolute ethanol (100 mL). Piperazine (2.61 g; 30.4 mmol) was dissolved in absolute ethanol (50 mL), and added dropwise at ambient temperature. After the complete addition, the yellow solution was allowed to stir at ambient temperature. After 4 h, the volatiles were completely removed in a vacuum, affording a viscous yellow oil. After standing at ambient conditions, a white crystalline product formed that was collected by filtration and washed with heptane. The solid was then collected and dried overnight in a vacuum oven at 70 °C. The yield was as follows: 18.4 g (76%); m.p. 125 °C. ^1^H-NMR (400.2 MHz, DMSO-*d*_6_) δ (ppm) was as follows: 4.24 (m, 16H, O–C**H**_2_–CH_3_); 3.99 (s, 8H, N–(C**H**_2_–C**H**_2_)_2_–N); 1.30 (t, *J*_H,H_ = 7.1 Hz, 24H, O–CH_2_–C**H**_3_). ^13^C-NMR (100.6 MHz, DMSO-*d*_6_) δ (ppm) was as follows: 170.5 (sdd, *J*_C,P_ = 260, 14.9 Hz, 4C, C-b); 161.8 (st, *J*_C,P_ = 18.3 Hz, 2C, C-a); 63.9 (tm ^a)^, 8C, O–**C**H_2_–CH_3_); 42.4 (t, 4C, N–(**C**H_2_–**C**H_2_)_2_–N); 16.2(tm ^a)^, 8C, O–CH_2_–**C**H_3_). ^31^P-NMR (162.0 MHz, DMSO-*d*_6_) δ (ppm) was as follows: 2.9. ^1^H-^13^C HMBC: O–C**H**_2_–CH_3_ → O–CH_2_–**C**H_3_; N–(C**H**_2_–C**H**_2_)_2_–N → C-a, N–(**C**H_2_–**C**H_2_)_2_–N; O–CH_2_C**H**_3_ → O–**C**H_2_–CH_3_. The following analysis was calculated for C_26_H_48_N_8_O_12_P_4_: C, 39.60; H, 6.14; N, 14.21. The following was found: C, 39.58; H, 6.04; N, 14.11.

^a)^ Because of steric reasons, a rather complicated signal pattern was observed for the P(=O)(OEt)_2_ carbons.

## 4. Conclusions

Cyanuric chloride was used as a starting material to synthesize bridged triazine-based organophosphorus compounds. The Michaelis–Arbuzov rearrangement of triethyl phosphite with cyanuric chloride afforded intermediate HEPT, which was used for the further substitution reactions. Because of the electron-withdrawing effect of the phosphonate group, the HEPT group showed a relative positive charge in the triazine ring, making the ring eligible for nucleophilic substitution reactions. This nature of HEPT was utilized to achieve a substitution reaction with ethylenediamine and piperazine at ambient temperature, affording EDA-bis-TEPT and Pip-bis-TEPT, respectively. Diethyl phosphite is the only by-product that was detected, and could be isolated by precipitating EDA-bis-TEPT and Pip-bis-TEPT in toluene. Single crystals for X-ray analysis provided compelling evidence regarding the chemical structures of the target compounds and the electronic distribution in a triazine ring. The correlation between the NMR spectroscopy and single crystal X-ray analysis could confirm the increase in the electron density of the triazine ring once one phosphonate group of HEPT was replaced with the amine group, resulting in either EDA-bis-TEPT or Pip-bis-TEPT. This change led to a decrease in the reactivity of the latter compounds to any further substitution reaction at ambient conditions, and prevented the formation of a mixture of substituted product or polymeric materials. However, more investigations are in progress in order to investigate the possibility of further substitution reactions, either at high temperatures or in different chemical solvents. The pyrolysis of the synthesized products showed the formation of the residual char, which is considered as a barrier to protect the sub-layers from heat and fire. An analysis of the volatiles that evolved upon pyrolysis showed the presence of diethyl phosphite, HPO, and H–C≡P, which are considered a suitable source for PO^•^. Such radical scavenging of the O^•^ and ^•^OH species does result in an additional protection of the matrix from fire. Therefore, these compounds have promising flame retardant characteristics [34]. Accordingly, the efficacy of these compounds as flame retardant additives will be further investigated in our future work.

## Figures and Tables

**Table 1:** Selected bond distances (Å) for the synthesized compounds 2,4,6-trisdiethoxyphosphinyl-1,3,5-triazine (HEPT); *N*,*N*′-Bis[4,6-bis(diethylphosphono)-1,3,5-Triazin-yl]-1,2-diaminoethane (EDA-bis-TEPT); *N*,*N*′-Bis[4,6-bis(diethylphosphono)-1,3,5-Triazin-yl]-piperazine (Pip-bis-TEPT).

HEPT		EDA-bis-TEPT		Pip-bis-TEPT	
Atoms	Distances (Å)	Atoms	Distances (Å)	Atoms	Distances (Å)
P1–C1	1.846 (4)	P1–C3	1.827 (5)	P1–C1	1.826 (3)
N1–C1	1.337 (6)	P2–C4	1.834 (6)	P2–C2	1.830 (3)
		N3–C3	1.355 (8)	N1–C1	1.344 (4)
		N3–C4	1.354 (7)	N1–C2	1.347 (4)
		N4–C4	1.323 (8)	N2–C2	1.323 (4)
		N4–C2	1.361 (8)	N2–C3	1.353 (4)
		N2–C3	1.309 (8)	N3–C1	1.326 (4)
		N2–C2	1.366 (7)	N3–C3	1.354 (4)
		N1–C2	1.335 (8)	N4–C3	1.343 (4)

**Table 2:** Thermal gravimetric analysis (TGA) data of the synthesized compounds.

Sample	T_onset_ (°C)		T_max_^a^ (°C)		Char Residue (wt%) at 800 °C		N:P (wt%)
	N_2_	O_2_	N_2_	O_2_	N_2_	O_2_	
HEPT	265 ± 1	265 ± 0.5	270	271	27 ± 0.5	20 ± 0.5	0.45
EDA-bis-TEPT	253 ± 0.5	252 ± 0.5	263	263	36 ± 1	25 ± 0.5	0.90
Pip-bis-TEPT	277 ± 3	261 ± 1	283	272	33 ± 1	25 ± 1	0.90

^a^ T_max_: the maximum-rate decomposition temperature.

**Table 3:** Elemental analysis of the char residue after TGA under air. EDX—energy-dispersive X-ray analysis.

Sample	EDX (wt%)			Elemental Loss (wt%) ^a^		
	P	C	N	P	C	N
HEPT	37 ± 2	24 ± 1	15 ± 1	61	87	65
EDA-bis-EPT	28 ± 2	26 ± 1	15 ± 0.5	57	83	74
Pip-bis-TEPT	27 ± 1	29 ± 2	18 ± 3	57	82	68

^a^ The elemental loss (wt%) was calculated based on the EDX analysis, from the following equation: (1 − ((char percentage x element percentage in char)/(100 × element percentage of the compound))) × 100.

**Table 4:** Crystal data and structure refinement of the synthesized substances.

Identification Code	HEPT	EDA-bis-TEPT	Pip-bis-TEPT
Empirical formula	C_15_H_30_N_3_O_9_P_3_	C_24_H_46_N_8_O_12_P_4_	C_26_H_48_N8O_12_P_4_
Formula weight	489.33	762.57	788.60
Temp/K	293	173	293
Crystal system	Hexagonal	Triclinic	Triclinic
Space group	P63/m	P-1	P-1
a (Å)	13.5150 (15)	7.463 (5)	7.3620 (9)
b (Å)	13.5150 (15)	21.171 (13)	11.8117 (16)
c (Å)	7.6602 (10)	21.200 (12)	12.4732 (16)
α (°)	90	118.49 (4)	106.602 (10)
β (°)	90	95.68 (5)	103.435 (10)
γ (°)	120	97.27 (5)	104.434 (10)
*V* (Å^3^)	1211.7 (4)	2858 (3)	951.2 (2)
*Z*	2	3	1
*D*_calc_ (g.cm^−3^)	1.341	1.329	1.377
Absorption coefficient (mm^−1^)	0.293	0.262	0.264
F(000)	516	1206	416
Crystal size /mm^3^	0.4 × 0.45 × 0.5	0.5 × 0.45 × 0.4	0.5 × 0.45 × 0.4
Radiation (Å)	MoKα (λ = 71073)	MoKα (λ = 0.71073)	MoKα (λ = 0.71073)
2θ range for data collection (°)	1.7 to 25.7	1.931 to 26.273	3.606 to 51.326
Index ranges	−16 ≤ h ≤ 14, −16 ≤ k ≤ 16, and −9 ≤ l ≤ 9	−8 ≤ h ≤ 9, −25 ≤ k ≤ 25, and −25 ≤ l ≤ 25	−8 ≤ h ≤ 8, −14 ≤ k ≤ 12, and −15 ≤ l ≤ 15
Reflections collected	5013	19228	8103
Independent reflections	5013 (R_int_ = 0.071)	10234 (R_int_ = 0.0906)	3528 (R_int_ = 0.0964)
Data/restraints/parameters	820/0/85	10,234/18/582	3528/0/225
Goodness of fit on F^2^	1.145	1.024	1.093
Final R indexes (I ≥ 2σ (I))	R_1_ = 0.0672, wR_2_ = 0.2142	R_1_ = 0.0947, wR_2_ = 0.2612	R_1_ = 0.0573, wR_2_ = 0.1588
Final R indexes (all data)	R_1_^a^ = 0.0853, wR_2_^b^ = 0.2379	R_1_^a^ = 0.1662, wR_2_^b^ = 0.3192	R_1_^a^ = 0.0700, wR_2_^b^ = 0.1804
Largest diff. peak/hole ( e Å^-3^)	0.64/−0.27	0.632/−0.793	0.97/−0.88

^a^ R_1_ = ∑(‖F_o_| − |Fcǁ)/∑|F_o_|. ^b^ wR_2_ = {[∑w(F_o_^2^ − F_c_^2^)^2^]/[ ∑w(F_o2_)^2^]}^1/2^, w = 1/[σ^2^(F_o_^2^) + (aP)^2^ + bP, where P = [max(F_o_^2^),0) + 2(F_c_^2^)]/3.

## Supplementary Materials

The following additional information is available online: Figure S1: ^1^H, ^13^C, and ^31^P-NMR spectra of HEPT (DMSO-*d*_6_); Figure S2A: ^1^H, ^13^C, and ^31^P-NMR spectra of EDA-bis-TEPT (DMSO-*d*_6_); Figure S2B: Regions of interest of ^1^H-^13^C HSQC (A) and ^1^H-^13^C HMBC (B) NMR spectra of EDA-bis-TEPT (DMSO-*d*_6_); Figure S3A: ^1^H, ^13^C, and ^31^P-NMR spectra of Pip-bis-TEPT (DMSO-*d*_6_); Figure S3B: Regions of interest of ^1^H-^13^C HSQC (A) and ^1^H-^13^C HMBC (B) NMR spectra of Pip-bis-TEPT (DMSO-*d*_6_); Figure S4: DSC curve of HEPT; Figure S5: DSC curve of EDA-bis-TEPT; Figure S6: DSC curve of Pip-bis-TEPT; Table S1: Final coordinates and equivalent isotropic displacement parameters of the non-hydrogen atoms for HEPT; Table S2: Hydrogen atom positions and isotropic displacement parameters for HEPT; Table S3: Anisotropic displacement parameters for HEPT; Table S4: Bond angles for HEPT; Table S5: Atomic coordinates and equivalent isotropic displacement parameters of the non-hydrogen atoms for EDA-bis-TEPT; Table S6: Hydrogen coordinates and isotropic displacement parameters for EDA-bis-TEPT; Table S7: Anisotropic displacement parameters for EDA-bis-TEPT; Table S8: Bond angles for EDA-bis-TEPT; Table S9: Fractional atomic coordinates and equivalent isotropic displacement parameters of the non-hydrogen atoms for Pip-bis-TEPT; Table S10: Hydrogen atom coordinates and isotropic displacement parameters for Pip-bis-TEPT; Table S11: Anisotropic displacement parameters for Pip-bis-TEPT; Table S12: Bond angles for Pip-bis-TEPT.
