# Supplementary material for: Insight into the Synthesis and Characterization of Organophosphorus-Based Bridged Triazine Compounds
Source: Molecules. 2019 Jul 23;24(14):2672. doi: 10.3390/molecules24142672 (PMC6681112; doi:10.3390/molecules24142672)
Supplement: Supplementary file 1 [file molecules-24-02672-s001.pdf]

Supplementary Materials

Insight into the Synthesis and Characterization of Organo-phosphorus based bridged Triazine compounds

Khalifah A. Salmeia [1,*], Antonia Neels [2,*], Dambarudhar Parida [1], Sandro Lehner [1], Daniel Rentsch [3] and Sabyasachi Gaan [1]

* Correspondence: khalifah.salmeia@empa.ch (K.A.S.); antonia.neels@empa.ch (A.N.).

Table of Contents

**Figure S1**. ^{1}H, ^{13}C and ^{31}P NMR spectra of HEPT (DMSO-d$_6$)	2
**Figure S2A**. ^{1}H, ^{13}C and ^{31}P NMR spectra of EDA-bis-TEPT (DMSO-d$_6$)	3
**Figure S2B**. Regions of interest of ^{1}H-^{13}C HSQC (A) and ^{1}H-^{13}C HMBC (B) NMR spectra of EDA-bis-TEPT (DMSO-d$_6$)	4
**Figure S3A**. ^{1}H, ^{13}C and ^{31}P NMR spectra of Pip-bis-TEPT (DMSO-d$_6$)	5
**Figure S3B**. Regions of interest of ^{1}H-^{13}C HSQC (A) and ^{1}H-^{13}C HMBC (B) NMR spectra of SKH-Pip-bis-TEPT (DMSO-d$_6$)	6
**Figure S4**. DSC curve of HEPT	7
**Figure S5**. DSC curve of EDA-bis-TEPT	7
**Figure S6**. DSC curve of Pip-bis-TEPT	7
**Table S1**. Final coordinates and equivalent isotropic displacement parameters of the non-hydrogen atoms for HEPT	8
**Table S2**. Hydrogen atom positions and isotropic displacement parameters for HEPT	8
**Table S3**. Anisotropic displacement parameters for HEPT	9
**Table S4**. Bond angles (°) for HEPT	9
**Table S5.** Atomic coordinates (Å×10^4) and equivalent isotropic displacement parameters (Å^2×10^3) of the non-hydrogen atoms for EDA-bis-TEPT	10
**Table S6**. Hydrogen coordinates (Å×10^4) and isotropic displacement parameters (Å^2×10^3) for EDA-bis-TEPT	12
**Table S7**. Anisotropic displacement parameters (Å^2×10^3) for EDA-bis-TEPT	15
**Table S8.** Bond angles (°) for EDA-bis-TEPT	17
**Table S9.** Fractional atomic coordinates (Å×10^4) and equivalent isotropic displacement parameters (Å^2×10^3) of the non-hydrogen atoms for Pip-bis-TEPT	23
**Table S10.** Hydrogen atom coordinates (Å×10^4) and isotropic displacement parameters (Å^{2}x10^3) for Pip-bis-TEPT	24
**Table S11.** Anisotropic displacement parameters (Å^{2}x10^3) for Pip-bis-TEPT	25
**Table S12.** Bond angles (°) for Pip-bis-TEPT	26

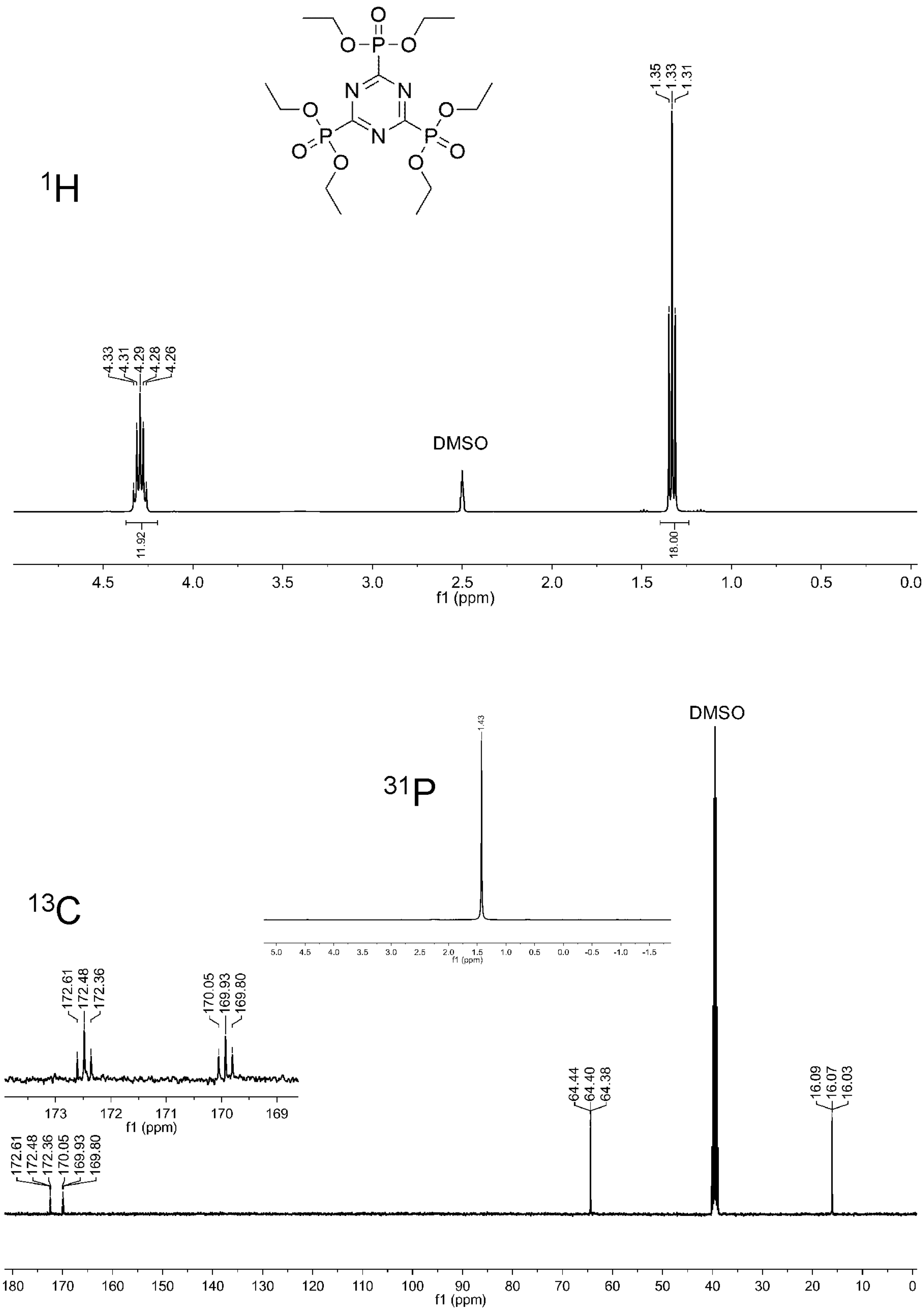

Figure S1. ^{1}H, ^{13}C and ^{31}P NMR spectra of HEPT (DMSO-d_6).

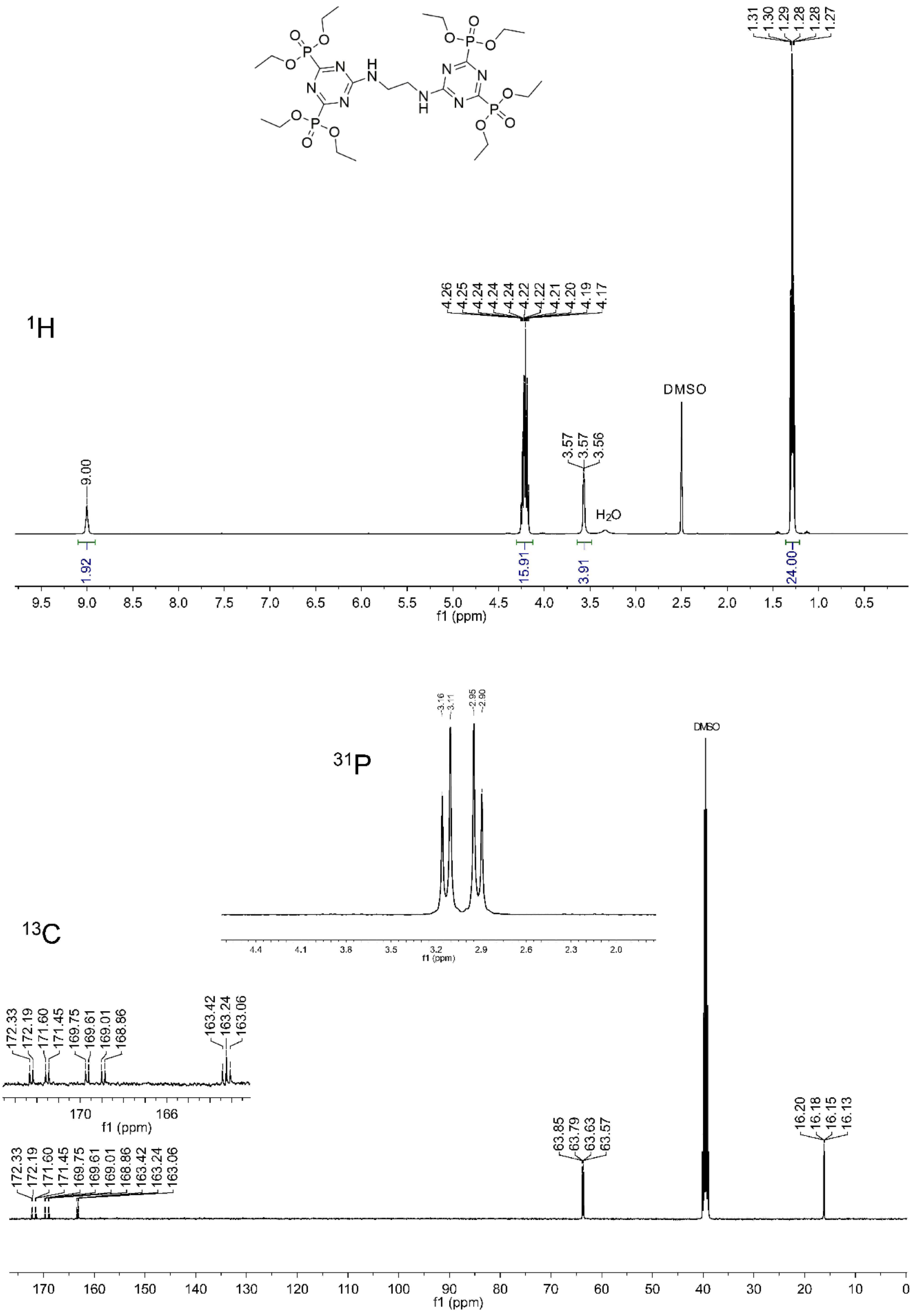

Figure S2A. ^{1}H, ^{13}C and ^{31}P NMR spectra of EDA-bis-TEPT (DMSO-d_6).

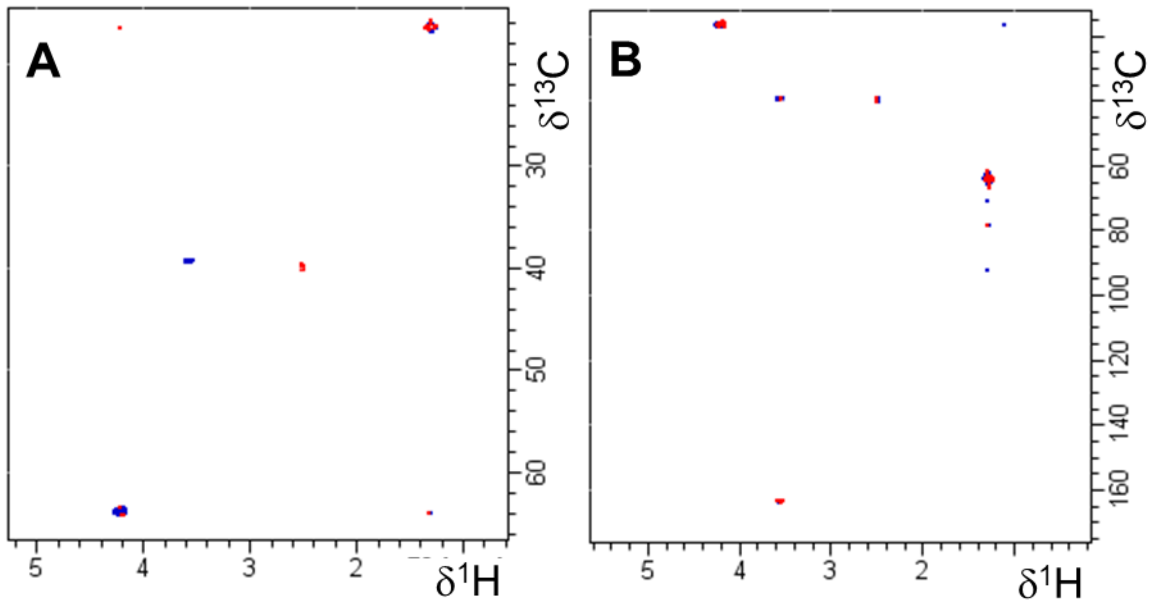

Figure S2B. Regions of interest of ^{1}H-^{13}C HSQC (A) and ^{1}H-^{13}C HMBC (B) NMR spectra of EDA-bis-TEPT (DMSO-d_6).

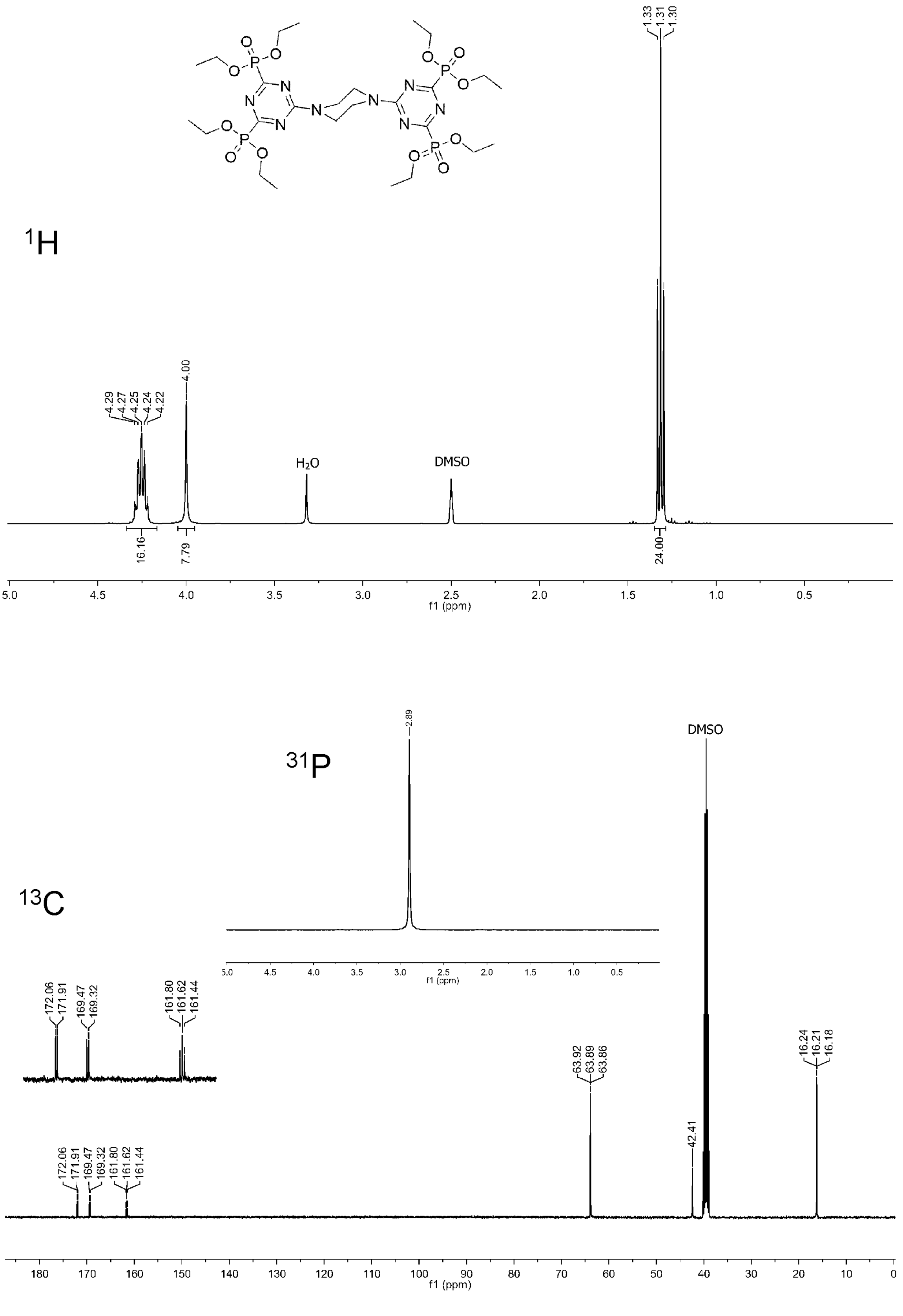

Figure S3A. ^{1}H, ^{13}C and ^{31}P NMR spectra of Pip-bis-TEPT (DMSO-d_6).

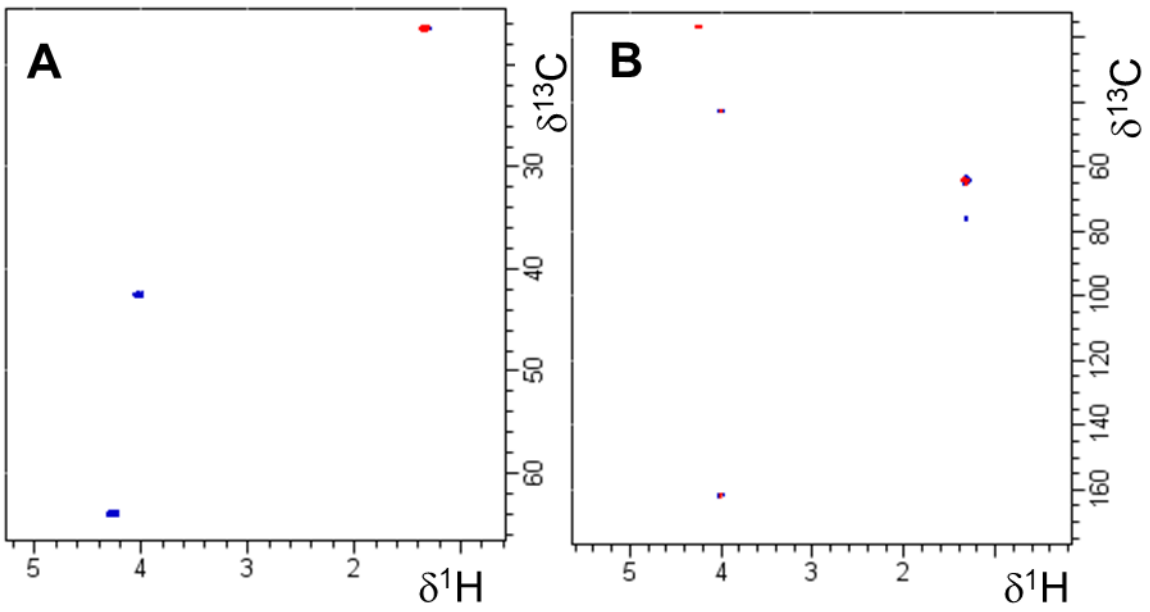

Figure S3B. Regions of interest of ^{1}H-^{13}C HSQC (A) and ^{1}H-^{13}C HMBC (B) NMR spectra of Pip-bis-TEPT (DMSO-d_6).

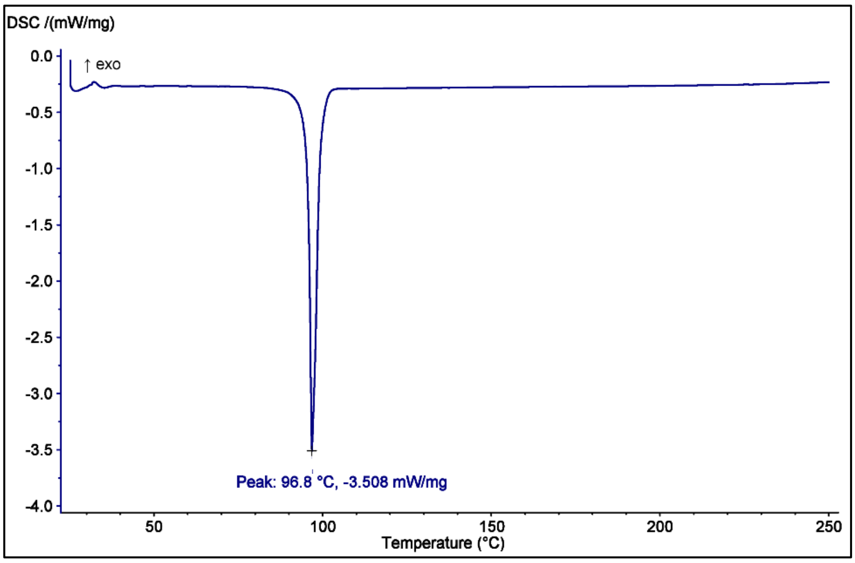

Figure S4. DSC curve of HEPT.

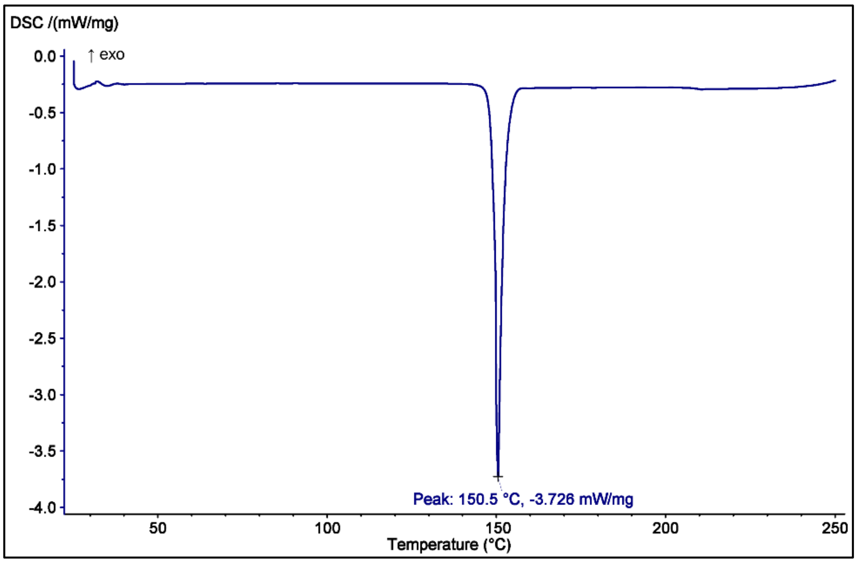

Figure S5. DSC curve of EDA-bis-TEPT.

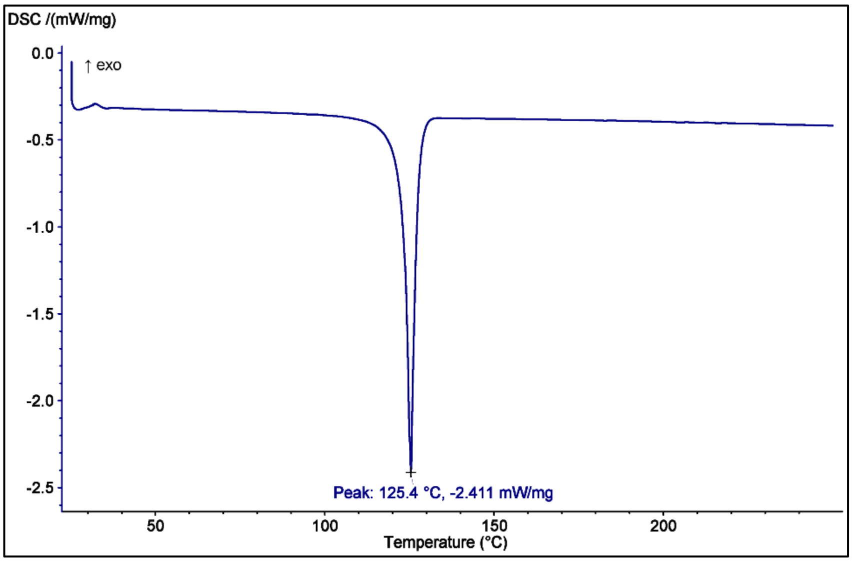

Figure S6. DSC curve of Pip-bis-TEPT.

Table S1. Final coordinates and equivalent isotropic displacement parameters of the non-hydrogen atoms for HEPT.

Atom	x	y	z	U(eq) [Å^2]
*P1	0.71483(12)	0.13060(12)	0.2863(4)	0.0680(11)
*O1	0.7177(4)	0.1100(4)	0.4726(6)	0.0877(19)
*O2	0.6217(4)	0.0316(3)	0.1751(6)	0.0767(14)
*O3	0.8224(4)	0.1620(4)	0.1775(5)	0.0818(14)
N1	0.5774(3)	0.2232(3)	1/4	0.0627(11)
C1	0.6868(3)	0.2494(3)	1/4	0.0603(12)
C2	0.5088(5)	-0.0557(6)	1/4	0.114(3)
*C3	0.5015(9)	-0.1569(7)	0.217(4)	0.122(10)
*C4	0.9375(5)	0.2310(6)	0.218(3)	0.081(6)
*C5	0.9969(7)	0.1673(7)	0.230(8)	0.104(10)

U(eq) = 1/3 of the trace of the orthogonalized U Tensor Starred Atom sites have a S.O.F less than 1.0

Table S2. Hydrogen atom positions and isotropic displacement parameters for HEPT.

Atom	x	y	z	U(iso) [Å^2]
*H2A	0.44670	-0.05120	0.19440	0.1360
*H2B	0.50630	-0.04440	0.37460	0.1360
*H3A	0.57570	-0.14940	0.22800	0.1470
*H3B	0.45000	-0.21290	0.29850	0.1470
*H3C	0.47360	-0.18070	0.10020	0.1470
*H4A	0.97350	0.28950	0.12920	0.0970
*H4B	0.94420	0.26880	0.32870	0.0970
*H5A	0.99520	0.14330	0.34790	0.1270
*H5B	1.07470	0.21490	0.19310	0.1270
*H5C	0.96020	0.10150	0.15510	0.1270

The Temperature Factor has the Form of Exp(-T) Where T = 8*(Pi**2)*U*(Sin(Theta)/Lambda)**2 for Isotropic Atoms.

Table S3. Anisotropic displacement parameters for HEPT.

Atom	U_{11} or U	U_{22}	U_{33}	U_{23}	U_{13}	U_{12}
P1	0.0647(9)	0.0564(9)	0.088(3)	0.0031(7)	0.0019(8)	0.0340(7)
O1	0.108(4)	0.086(3)	0.083(3)	0.012(2)	-0.006(3)	0.059(3)
O2	0.069(2)	0.055(2)	0.100(3)	-0.0032(18)	0.0018(19)	0.0265(19)
O3	0.066(2)	0.070(2)	0.116(3)	-.005(2)	0.010(2)	0.039(2)
N1	0.0531(18)	0.0548(18)	0.079(2)	0	0	0.0261(15)
C1	0.058(2)	0.058(2)	0.066(2)	0	0	0.0298(18)
C2	0.086(4)	0.100(4)	0.154(6)	0	0	0.045(3)
C3	0.142(7)	0.069(4)	0.13(3)	-0.014(7)	-0.023(10)	0.033(4)
C4	0.065(3)	0.072(3)	0.105(16)	0.007(4)	0.006(4)	0.034(3)
C5	0.084(4)	0.090(4)	0.15(3)	-0.021(14)	-0.003(10)	0.053(3)

The Temperature Factor has the Form of Exp(-T) Where T = 8*(Pi**2)*U*(Sin(Theta)/Lambda)**2.

For Isotropic Atoms T = 2*(Pi**2)*Sumij(h(i)*h(j)*U(i,j)*Astar(i)*Astar(j)).

For Anisotropic Atoms. Astar(i) are Reciprocal Axial Lengths and h(i) are the Reflection Indices.

Table S4. Bond angles (°) for HEPT.

O1-P1-O2	117.4(3)
O1-P1-O3	118.1(3)
O1-P1-C1	110.5(2)
O2-P1-O3	100.8(3)
O2-P1-C1	103.7(2)
O3-P1-C1	104.8(2)
P1-O2-C2	122.3(3)
P1-O3-C4	130.5(9)
P1-C1-N1	116.8(3)
O2-C2-C3	104.9(9)
C2-C3-H3A	110.00
C2-C3-H3B	110.00
O3-C4-C5	112.5(8)

Table S5. Atomic coordinates (Å×10^4) and equivalent isotropic displacement parameters (Å^2×10^3) of the non-hydrogen atoms for EDA-bis-TEPT. U(eq) is defined as one third of the trace of the orthogonalized U_{ij} tensor.

Atom	x	y	z	U(eq)
C(1)	-4129(11)	9863(4)	9983(4)	78(2)
C(2)	-4728(8)	8580(3)	9729(3)	53(2)
C(3)	-5188(8)	7427(3)	9533(3)	52(2)
C(4)	-5783(8)	7626(3)	8582(3)	48(1)
O(2)	-3642(9)	7109(4)	10523(4)	110(2)
C(5)	-3610(30)	6935(11)	11106(11)	105(2)
C(5A)	-3590(30)	7531(11)	11225(11)	105(2)
C(6)	-1970(30)	7164(11)	11535(12)	105(2)
C(6A)	-2510(30)	7459(11)	11758(11)	105(2)
O(3)	-4732(9)	6089(3)	9327(3)	96(2)
C(7)	-5560(30)	5486(14)	8683(18)	105(2)
C(7A)	-6190(30)	5515(14)	8712(18)	105(2)
C(8)	-5640(50)	4830(14)	8720(14)	105(2)
C(8A)	-5900(50)	4792(13)	8495(14)	105(2)
C(9)	-6080(30)	5842(18)	7023(19)	87(5)
C(9A)	-6870(30)	5894(18)	6977(19)	87(5)
C(10)	-7080(30)	5427(15)	6230(16)	87(5)
C(10A)	-6340(30)	5305(15)	6322(16)	87(5)
C(11)	-5890(30)	8445(11)	7492(11)	96(4)
C(11A)	-6170(30)	8039(11)	6923(11)	96(4)
C(12)	-5480(60)	8494(16)	6821(14)	96(4)
C(12A)	-5390(60)	8707(15)	7045(14)	96(4)
C(13)	-880(11)	9981(4)	5118(4)	73(2)
C(14)	-549(9)	9732(3)	6154(4)	57(2)
C(15)	-651(8)	8581(3)	5963(3)	49(1)
C(16)	-277(8)	9531(3)	7105(3)	49(1)
C(17)	-1640(30)	7486(11)	4041(9)	95(3)
C(17A)	-1900(30)	6925(11)	3893(9)	95(3)
C(18)	-2850(50)	6816(13)	3331(15)	95(3)
C(18A)	-2450(50)	7047(13)	3335(15)	95(3)
O(9)	-2120(8)	7193(3)	5605(3)	87(2)
C(19)	-1820(40)	6995(13)	6120(20)	107(4)
C(19A)	-1240(40)	6978(15)	6130(20)	107(4)
C(20)	-1910(50)	6229(12)	5802(11)	107(4)
C(20A)	-2150(50)	6324(13)	6065(12)	107(4)
C(21)	-100(30)	11226(11)	8679(11)	103(1)
C(21A)	-500(30)	11144(12)	9173(11)	103(1)
C(22)	-1680(30)	11606(12)	9175(11)	103(1)
C(22A)	-640(30)	11728(12)	9432(11)	103(1)
O(12)	-958(9)	9328(4)	8235(3)	103(1)
C(23)	-820(30)	8624(13)	8154(10)	103(1)

C(23A)	-40(30)	8749(13)	8235(11)	103(1)
C(24)	-610(50)	8710(13)	8889(10)	103(1)
C(24A)	-640(50)	8514(13)	8718(11)	103(1)
C(25)	-8990(10)	5128(4)	132(4)	75(2)
C(26)	-8303(9)	6152(3)	1422(4)	55(2)
C(27)	-8387(8)	5966(3)	2379(3)	46(1)
C(28)	-7620(8)	7103(3)	2571(3)	50(1)
C(29)	-9350(30)	4047(9)	1569(10)	91(1)
C(29A)	-9190(30)	3888(9)	1985(10)	91(1)
C(30)	-8800(30)	3341(11)	1510(11)	91(1)
C(30A)	-9230(30)	3313(10)	1258(11)	91(1)
C(31)	-7293(14)	6134(5)	4142(5)	91(1)
C(32)	-7086(13)	5911(5)	4655(5)	91(1)
C(33)	-6200(30)	8677(10)	2446(12)	105(1)
C(33A)	-5450(30)	9114(10)	3002(13)	105(1)
C(34)	-4940(30)	9315(11)	2535(12)	105(1)
C(34A)	-4150(30)	9368(11)	2829(13)	105(1)
C(35)	-6369(15)	8197(5)	4498(6)	105(1)
C(36)	-5583(14)	8809(5)	5189(5)	105(1)
N(1)	-4150(9)	9302(3)	10194(3)	74(2)
N(2)	-4632(7)	8128(3)	10017(3)	59(1)
N(3)	-5763(7)	7122(3)	8801(3)	50(1)
N(4)	-5313(7)	8350(3)	9009(3)	55(1)
N(5)	-648(9)	10200(3)	5893(3)	75(2)
N(6)	-679(8)	9008(3)	5657(3)	57(1)
N(7)	-453(7)	8799(3)	6674(3)	51(1)
N(8)	-352(7)	10018(3)	6887(3)	57(1)
N(9)	-8449(9)	5890(3)	690(3)	77(2)
N(10)	-8670(7)	5660(3)	1650(3)	55(1)
N(11)	-7882(7)	6673(3)	2874(3)	51(1)
N(12)	-7765(7)	6883(3)	1869(3)	54(1)
O(1)	-7078(8)	6703(4)	10063(4)	103(2)
O(4)	-8524(7)	7156(3)	7435(3)	91(2)
O(5)	-5703(8)	6587(3)	7191(3)	86(2)
O(6)	-5447(8)	7830(3)	7443(3)	82(2)
O(7)	950(8)	7432(3)	5283(3)	91(2)
O(8)	-2108(8)	7435(3)	4613(3)	82(2)
O(10)	2141(10)	10057(4)	8363(3)	103(1)
O(11)	-873(9)	10514(4)	8406(3)	103(1)
O(13)	-10679(9)	5276(3)	2849(3)	91(1)
O(14)	-8274(9)	4613(3)	2176(3)	91(1)
O(15)	-7268(9)	5602(3)	3407(3)	91(1)

O(16)	-8755(10)	8364(3)	3311(4)	105(1)
O(17)	-5720(9)	8415(3)	2903(4)	105(1)
O(18)	-5821(9)	8235(3)	3903(4)	105(1)
P(1)	-5327(3)	6791(1)	9885(1)	68(1)
P(2)	-6582(3)	7268(1)	7602(1)	60(1)
P(3)	-811(3)	7601(1)	5332(1)	61(1)
P(4)	209(3)	9887(1)	8093(1)	68(1)
P(6)	-7115(3)	8093(1)	3206(1)	67(1)
P(5)	-8847(3)	5331(1)	2730(1)	60(1)

Table S6. Hydrogen coordinates (Å×10[4]) and isotropic displacement parameters (Å[2]×10[3]) for EDA-bis-TEPT.

	x	y	z	U(eq)
H(1A)	-3884	9656	9478	93
H(1B)	-3109	10283	10312	93
H(5A)	-4519	7165	11408	127
H(5B)	-3976	6395	10886	127
H(5C)	-4872	7477	11305	127
H(5D)	-3206	8045	11338	127
H(6A)	-1196	6803	11307	158
H(6B)	-2115	7217	12012	158
H(6C)	-1389	7640	11605	158
H(6D)	-2792	7779	12242	158
H(6E)	-1206	7601	11761	158
H(6F)	-2778	6946	11645	158
H(7A)	-4876	5451	8294	127
H(7B)	-6823	5537	8550	127
H(7C)	-7401	5563	8864	127
H(7D)	-6211	5593	8287	127
H(8A)	-4432	4698	8702	158
H(8B)	-6541	4430	8304	158
H(8C)	-6019	4911	9179	158
H(8D)	-5796	4724	8922	158
H(8E)	-4769	4724	8294	158
H(8F)	-6944	4430	8121	158
H(9B)	-6857	5780	7348	104
H(9A)	-4923	5670	7072	104
H(9C)	-8168	5916	6863	104
H(9D)	-6727	5788	7384	104
H(10A)	-7793	4952	6119	131
H(10B)	-6189	5345	5903	131
H(10C)	-7917	5718	6157	131
H(10D)	-7268	4850	6116	131

H(10E)	-5144	5218	6463	131
H(10F)	-6258	5456	5954	131
H(11A)	-5162	8882	7944	115
H(11B)	-7206	8438	7516	115
H(11C)	-6032	7664	6427	115
H(11D)	-7501	8022	6922	115
H(12A)	-4345	8319	6690	143
H(12B)	-5337	9008	6935	143
H(12C)	-6501	8189	6408	143
H(12D)	-5593	9089	7518	143
H(12E)	-5938	8782	6653	143
H(12F)	-4064	8736	7052	143
H(13A)	-1602	9471	4820	87
H(13B)	-1579	10308	5032	87
H(17A)	-319	7475	4031	114
H(17B)	-1861	7954	4080	114
H(17C)	-587	6897	3906	114
H(17D)	-2586	6434	3765	114
H(18A)	-4118	6888	3296	143
H(18B)	-2815	6364	3349	143
H(18C)	-2370	6775	2901	143
H(18D)	-3691	7157	3358	143
H(18E)	-2469	6607	2864	143
H(18F)	-1599	7465	3383	143
H(19A)	-2742	7142	6439	129
H(19B)	-587	7260	6427	129
H(19C)	34	6930	6058	129
H(19D)	-1173	7382	6641	129
H(20A)	-1600	6114	6189	161
H(20B)	-1036	6077	5465	161
H(20C)	-3157	5966	5531	161
H(20D)	-3427	6359	6127	161
H(20E)	-1533	6243	6441	161
H(20F)	-2140	5912	5578	161
H(21A)	-6	11352	8289	123
H(21B)	1125	11364	8988	123
H(21C)	-1205	10949	9435	123
H(21D)	809	11177	9350	123
H(22A)	-2892	11410	8858	154
H(22B)	-1377	12141	9388	154
H(22C)	-1684	11488	9568	154
H(22D)	-974	11849	9050	154
H(22E)	522	12048	9748	154

H(22F)	-1611	11803	9728	154
H(23A)	263	8459	7931	123
H(23B)	-1932	8252	7834	123
H(23C)	-288	8320	7731	123
H(23D)	1303	8939	8380	123
H(24A)	-1806	8728	9045	154
H(24B)	263	9167	9236	154
H(24C)	-155	8291	8877	154
H(24D)	19	8135	8701	154
H(24E)	-1964	8309	8567	154
H(24F)	-388	8935	9219	154
H(25A)	-8542	4825	328	90
H(25B)	-8387	5046	-289	90
H(29A)	-9149	4083	1132	109
H(29B)	-10663	4042	1608	109
H(29C)	-8583	3756	2329	109
H(29D)	-10472	3908	2063	109
H(30A)	-8786	3359	1981	137
H(30B)	-7577	3309	1382	137
H(30C)	-9695	2907	1129	137
H(30D)	-9140	2857	1260	137
H(30E)	-8199	3447	1066	137
H(30F)	-10391	3239	946	137
H(31B)	-6300	6565	4295	109
H(31A)	-8473	6303	4153	109
H(32A)	-7010	6333	5147	137
H(32B)	-5958	5715	4634	137
H(32C)	-8144	5527	4549	137
H(33A)	-7421	8810	2518	127
H(33B)	-6343	8272	1933	127
H(33C)	-6578	9144	2738	127
H(33D)	-5401	9456	3531	127
H(34A)	-4682	9706	3051	158
H(34B)	-5494	9494	2231	158
H(34C)	-3785	9170	2384	158
H(34D)	-3001	9405	3124	158
H(34E)	-4266	9858	2916	158
H(34F)	-4137	9043	2308	158
H(35A)	-6051	7751	4484	127
H(35B)	-7723	8139	4439	127
H(36A)	-6192	8776	5564	127
H(36B)	-5740	9262	5188	127

H(36C)	-4271	8817	5297	127
H(1N)	-3755	9448	10658	89
H(5N)	-566	10669	6214	90
H(9N)	-8192	6212	547	92

Table S7. Anisotropic displacement parameters (Å^2×10^3) for EDA-bis-TEPT. The anisotropic displacement factor exponent takes the form: -2 π^2[h^2 a*^{2}U$_{11}$ + ... +2 h ka* b* U$_{12}$].

Atom	U11	U22	U33	U23	U13	U12
C(1)	107(6)	46(4)	63(4)	25(4)	-15(4)	-9(4)
C(2)	55(4)	48(3)	47(4)	23(3)	-2(3)	1(3)
C(3)	57(4)	56(4)	51(4)	34(3)	5(3)	5(3)
C(4)	49(3)	52(3)	50(4)	32(3)	8(3)	7(3)
O(2)	129(5)	122(5)	95(5)	77(4)	-20(4)	9(4)
C(5)	132(7)	88(4)	96(6)	47(4)	2(5)	34(4)
C(5A)	132(7)	88(4)	96(6)	47(4)	2(5)	34(4)
C(6)	132(7)	88(4)	96(6)	47(4)	2(5)	34(4)
C(6A)	132(7)	88(4)	96(6)	47(4)	2(5)	34(4)
O(3)	129(5)	71(3)	94(4)	47(4)	6(4)	24(3)
C(7)	132(7)	88(4)	96(6)	47(4)	2(5)	34(4)
C(7A)	132(7)	88(4)	96(6)	47(4)	2(5)	34(4)
C(8)	132(7)	88(4)	96(6)	47(4)	2(5)	34(4)
C(8A)	132(7)	88(4)	96(6)	47(4)	2(5)	34(4)
C(9)	110(16)	76(7)	78(6)	39(4)	33(12)	12(10)
C(9A)	110(16)	76(7)	78(6)	39(4)	33(12)	12(10)
C(10)	110(16)	76(7)	78(6)	39(4)	33(12)	12(10)
C(10A)	110(16)	76(7)	78(6)	39(4)	33(12)	12(10)
C(11)	109(6)	103(11)	93(9)	76(9)	-8(9)	-10(8)
C(11A)	109(6)	103(11)	93(9)	76(9)	-8(9)	-10(8)
C(12)	109(6)	103(11)	93(9)	76(9)	-8(9)	-10(8)
C(12A)	109(6)	103(11)	93(9)	76(9)	-8(9)	-10(8)
C(13)	112(6)	55(4)	63(5)	36(4)	14(4)	32(4)
C(14)	72(4)	50(4)	56(4)	28(4)	16(3)	28(3)
C(15)	52(3)	43(3)	44(4)	16(3)	8(3)	13(3)
C(16)	60(4)	43(3)	36(3)	13(3)	12(3)	16(3)
C(17)	133(9)	86(8)	52(4)	23(7)	13(5)	30(8)
C(17A)	133(9)	86(8)	52(4)	23(7)	13(5)	30(8)
C(18)	133(9)	86(8)	52(4)	23(7)	13(5)	30(8)
C(18A)	133(9)	86(8)	52(4)	23(7)	13(5)	30(8)
O(9)	121(4)	61(3)	72(3)	35(3)	1(3)	2(3)
C(19)	131(9)	91(5)	92(6)	63(5)	-40(9)	-33(6)
C(19A)	131(9)	91(5)	92(6)	63(5)	-40(9)	-33(6)
C(20)	131(9)	91(5)	92(6)	63(5)	-40(9)	-33(6)
C(20A)	131(9)	91(5)	92(6)	63(5)	-40(9)	-33(6)

C(21)	128(3)	102(2)	67(2)	33(2)	20(2)	27(2)
C(21A)	128(3)	102(2)	67(2)	33(2)	20(2)	27(2)
C(22)	128(3)	102(2)	67(2)	33(2)	20(2)	27(2)
C(22A)	128(3)	102(2)	67(2)	33(2)	20(2)	27(2)
O(12)	128(3)	102(2)	67(2)	33(2)	20(2)	27(2)
C(23)	128(3)	102(2)	67(2)	33(2)	20(2)	27(2)
C(23A)	128(3)	102(2)	67(2)	33(2)	20(2)	27(2)
C(24)	128(3)	102(2)	67(2)	33(2)	20(2)	27(2)
C(24A)	128(3)	102(2)	67(2)	33(2)	20(2)	27(2)
C(25)	102(5)	70(5)	51(4)	30(4)	25(4)	4(4)
C(26)	71(4)	49(4)	56(4)	32(3)	24(3)	12(3)
C(27)	54(3)	39(3)	51(4)	23(3)	23(3)	13(2)
C(28)	55(3)	40(3)	54(4)	24(3)	12(3)	7(3)
C(29)	131(2)	70(2)	91(2)	48(2)	40(2)	33(2)
C(29A)	131(2)	70(2)	91(2)	48(2)	40(2)	33(2)
C(30)	131(2)	70(2)	91(2)	48(2)	40(2)	33(2)
C(30A)	131(2)	70(2)	91(2)	48(2)	40(2)	33(2)
C(31)	131(2)	70(2)	91(2)	48(2)	40(2)	33(2)
C(32)	131(2)	70(2)	91(2)	48(2)	40(2)	33(2)
C(33)	128(3)	69(2)	104(3)	38(2)	20(2)	-2(2)
C(33A)	128(3)	69(2)	104(3)	38(2)	20(2)	-2(2)
C(34)	128(3)	69(2)	104(3)	38(2)	20(2)	-2(2)
C(34A)	128(3)	69(2)	104(3)	38(2)	20(2)	-2(2)
C(35)	128(3)	69(2)	104(3)	38(2)	20(2)	-2(2)
C(36)	128(3)	69(2)	104(3)	38(2)	20(2)	-2(2)
N(1)	104(5)	53(3)	48(3)	22(3)	-13(3)	-1(3)
N(2)	65(3)	59(3)	54(3)	34(3)	0(3)	2(3)
N(3)	60(3)	44(3)	44(3)	23(3)	5(2)	6(2)
N(4)	71(3)	51(3)	42(3)	25(3)	5(2)	2(2)
N(5)	125(5)	48(3)	64(4)	30(3)	26(4)	38(3)
N(6)	80(4)	47(3)	44(3)	21(3)	15(3)	23(3)
N(7)	67(3)	46(3)	41(3)	20(3)	12(2)	15(2)
N(8)	74(4)	47(3)	43(3)	16(3)	13(3)	14(2)

Table S8. Bond angles (°) for EDA-bis-TEPT.

N(1)-C(1)-C(1)#1	113.4(8)	C(5)-C(6)-H(6C)	109.5
N(1)-C(1)-H(1A)	108.9	H(6A)-C(6)-H(6C)	109.5
C(1)#1-C(1)-H(1A)	108.9	H(6B)-C(6)-H(6C)	109.5
N(1)-C(1)-H(1B)	108.9	C(5A)-C(6A)-H(6D)	109.5
C(1)#1-C(1)-H(1B)	108.9	C(5A)-C(6A)-H(6E)	109.5
H(1A)-C(1)-H(1B)	107.7	H(6D)-C(6A)-H(6E)	109.5
N(1)-C(2)-N(4)	118.1(5)	C(5A)-C(6A)-H(6F)	109.5
N(1)-C(2)-N(2)	116.8(5)	H(6D)-C(6A)-H(6F)	109.5
N(4)-C(2)-N(2)	125.1(5)	H(6E)-C(6A)-H(6F)	109.5
N(2)-C(3)-N(3)	127.1(5)	C(7)-O(3)-P(1)	134.9(11)
N(2)-C(3)-P(1)	116.6(4)	C(7A)-O(3)-P(1)	116.6(10)
N(3)-C(3)-P(1)	116.2(4)	O(3)-C(7)-C(8)	112(2)
N(4)-C(4)-N(3)	126.2(5)	O(3)-C(7)-H(7A)	109.3
N(4)-C(4)-P(2)	117.1(4)	C(8)-C(7)-H(7A)	109.3
N(3)-C(4)-P(2)	116.7(4)	O(3)-C(7)-H(7B)	109.3
C(5A)-O(2)-P(1)	128.1(11)	C(8)-C(7)-H(7B)	109.3
C(5)-O(2)-P(1)	121.8(9)	H(7A)-C(7)-H(7B)	107.9
C(6)-C(5)-O(2)	113.2(18)	C(8A)-C(7A)-O(3)	111.9(17)
C(6)-C(5)-H(5A)	108.9	C(8A)-C(7A)-H(7C)	109.2
O(2)-C(5)-H(5A)	108.9	O(3)-C(7A)-H(7C)	109.2
C(6)-C(5)-H(5B)	108.9	C(8A)-C(7A)-H(7D)	109.2
O(2)-C(5)-H(5B)	108.9	O(3)-C(7A)-H(7D)	109.2
H(5A)-C(5)-H(5B)	107.8	H(7C)-C(7A)-H(7D)	107.9
O(2)-C(5A)-C(6A)	122.5(17)	C(7)-C(8)-H(8A)	109.5
O(2)-C(5A)-H(5C)	106.7	C(7)-C(8)-H(8B)	109.5
C(6A)-C(5A)-H(5C)	106.7	H(8A)-C(8)-H(8B)	109.5
O(2)-C(5A)-H(5D)	106.7	C(7)-C(8)-H(8C)	109.5
C(6A)-C(5A)-H(5D)	106.7	H(8A)-C(8)-H(8C)	109.5
H(5C)-C(5A)-H(5D)	106.6	H(8B)-C(8)-H(8C)	109.5
C(5)-C(6)-H(6A)	109.5	C(7A)-C(8A)-H(8D)	109.5
C(5)-C(6)-H(6B)	109.5	C(7A)-C(8A)-H(8E)	109.5
H(6A)-C(6)-H(6B)	109.5	H(8D)-C(8A)-H(8E)	109.5

C(7A)-C(8A)-H(8F)	109.5	C(12)-C(11)-H(11B)	109.8
H(8D)-C(8A)-H(8F)	109.5	H(11A)-C(11)-H(11B)	108.2
H(8E)-C(8A)-H(8F)	109.5	C(12A)-C(11A)-O(6)	116.3(19)
O(5)-C(9)-C(10)	103(2)	C(12A)-C(11A)-H(11C)	108.2
O(5)-C(9)-H(9B)	111.1	O(6)-C(11A)-H(11C)	108.2
C(10)-C(9)-H(9B)	111.1	C(12A)-C(11A)-H(11D)	108.2
O(5)-C(9)-H(9A)	111.1	O(6)-C(11A)-H(11D)	108.2
C(10)-C(9)-H(9A)	111.1	H(11C)-C(11A)-H(11D)	107.4
H(9B)-C(9)-H(9A)	109.0	C(11)-C(12)-H(12A)	109.5
O(5)-C(9A)-C(10A)	109(2)	C(11)-C(12)-H(12B)	109.5
O(5)-C(9A)-H(9C)	109.9	H(12A)-C(12)-H(12B)	109.5
C(10A)-C(9A)-H(9C)	109.9	C(11)-C(12)-H(12C)	109.5
O(5)-C(9A)-H(9D)	109.9	H(12A)-C(12)-H(12C)	109.5
C(10A)-C(9A)-H(9D)	109.9	H(12B)-C(12)-H(12C)	109.5
H(9C)-C(9A)-H(9D)	108.3	C(11A)-C(12A)-H(12D)	109.5
C(9)-C(10)-H(10A)	109.5	C(11A)-C(12A)-H(12E)	109.5
C(9)-C(10)-H(10B)	109.5	H(12D)-C(12A)-H(12E)	109.5
H(10A)-C(10)-H(10B)	109.5	C(11A)-C(12A)-H(12F)	109.5
C(9)-C(10)-H(10C)	109.5	H(12D)-C(12A)-H(12F)	109.5
H(10A)-C(10)-H(10C)	109.5	H(12E)-C(12A)-H(12F)	109.5
H(10B)-C(10)-H(10C)	109.5	C(13)#2-C(13)-N(5)	111.8(8)
C(9A)-C(10A)-H(10D)	109.5	C(13)#2-C(13)-H(13A)	109.3
C(9A)-C(10A)-H(10E)	109.5	N(5)-C(13)-H(13A)	109.3
H(10D)-C(10A)-H(10E)	109.5	C(13)#2-C(13)-H(13B)	109.3
C(9A)-C(10A)-H(10F)	109.5	N(5)-C(13)-H(13B)	109.3
H(10D)-C(10A)-H(10F)	109.5	H(13A)-C(13)-H(13B)	107.9
H(10E)-C(10A)-H(10F)	109.5	N(8)-C(14)-N(5)	117.6(6)
O(6)-C(11)-C(12)	110(2)	N(8)-C(14)-N(6)	124.8(5)
O(6)-C(11)-H(11A)	109.8	N(5)-C(14)-N(6)	117.5(6)
C(12)-C(11)-H(11A)	109.8	N(7)-C(15)-N(6)	127.1(5)
O(6)-C(11)-H(11B)	109.8	N(7)-C(15)-P(3)	116.9(4)
N(6)-C(15)-P(3)	116.0(4)	C(20)-C(19)-H(19A)	109.2
N(8)-C(16)-N(7)	126.8(5)	O(9)-C(19)-H(19B)	109.2

N(8)-C(16)-P(4)	117.1(4)	C(20)-C(19)-H(19B)	109.2
N(7)-C(16)-P(4)	116.0(4)	H(19A)-C(19)-H(19B)	107.9
O(8)-C(17)-C(18)	107.2(18)	C(20A)-C(19A)-O(9)	115.1(15)
O(8)-C(17)-H(17A)	110.3	C(20A)-C(19A)-H(19C)	108.5
C(18)-C(17)-H(17A)	110.3	O(9)-C(19A)-H(19C)	108.5
O(8)-C(17)-H(17B)	110.3	C(20A)-C(19A)-H(19D)	108.5
C(18)-C(17)-H(17B)	110.3	O(9)-C(19A)-H(19D)	108.5
H(17A)-C(17)-H(17B)	108.5	H(19C)-C(19A)-H(19D)	107.5
C(18A)-C(17A)-O(8)	118.7(19)	C(19)-C(20)-H(20A)	109.5
C(18A)-C(17A)-H(17C)	107.6	C(19)-C(20)-H(20B)	109.5
O(8)-C(17A)-H(17C)	107.6	H(20A)-C(20)-H(20B)	109.5
C(18A)-C(17A)-H(17D)	107.6	C(19)-C(20)-H(20C)	109.5
O(8)-C(17A)-H(17D)	107.6	H(20A)-C(20)-H(20C)	109.5
H(17C)-C(17A)-H(17D)	107.1	H(20B)-C(20)-H(20C)	109.5
C(17)-C(18)-H(18A)	109.5	C(19A)-C(20A)-H(20D)	109.5
C(17)-C(18)-H(18B)	109.5	C(19A)-C(20A)-H(20E)	109.5
H(18A)-C(18)-H(18B)	109.5	H(20D)-C(20A)-H(20E)	109.5
C(17)-C(18)-H(18C)	109.5	C(19A)-C(20A)-H(20F)	109.5
H(18A)-C(18)-H(18C)	109.5	H(20D)-C(20A)-H(20F)	109.5
H(18B)-C(18)-H(18C)	109.5	H(20E)-C(20A)-H(20F)	109.5
C(17A)-C(18A)-H(18D)	109.5	O(11)-C(21)-C(22)	98.3(15)
C(17A)-C(18A)-H(18E)	109.5	O(11)-C(21)-H(21A)	112.1
H(18D)-C(18A)-H(18E)	109.5	C(22)-C(21)-H(21A)	112.1
C(17A)-C(18A)-H(18F)	109.5	O(11)-C(21)-H(21B)	112.1
H(18D)-C(18A)-H(18F)	109.5	C(22)-C(21)-H(21B)	112.1
H(18E)-C(18A)-H(18F)	109.5	H(21A)-C(21)-H(21B)	109.8
C(19)-O(9)-P(3)	132.1(12)	C(22A)-C(21A)-O(11)	135(2)
C(19A)-O(9)-P(3)	117.3(11)	C(22A)-C(21A)-H(21C)	103.5
O(9)-C(19)-C(20)	112(2)	O(11)-C(21A)-H(21C)	103.6
O(9)-C(19)-H(19A)	109.2	C(22A)-C(21A)-H(21D)	103.6
O(11)-C(21A)-H(21D)	103.6	H(24A)-C(24)-H(24C)	109.5
H(21C)-C(21A)-H(21D)	105.3	H(24B)-C(24)-H(24C)	109.5
C(21)-C(22)-H(22A)	109.5	C(23A)-C(24A)-H(24D)	109.5

C(21)-C(22)-H(22B)	109.5	C(23A)-C(24A)-H(24E)	109.5
H(22A)-C(22)-H(22B)	109.5	H(24D)-C(24A)-H(24E)	109.5
C(21)-C(22)-H(22C)	109.5	C(23A)-C(24A)-H(24F)	109.5
H(22A)-C(22)-H(22C)	109.5	H(24D)-C(24A)-H(24F)	109.5
H(22B)-C(22)-H(22C)	109.5	H(24E)-C(24A)-H(24F)	109.5
C(21A)-C(22A)-H(22D)	109.5	N(9)-C(25)-C(25)#3	113.9(9)
C(21A)-C(22A)-H(22E)	109.5	N(9)-C(25)-H(25A)	108.8
H(22D)-C(22A)-H(22E)	109.5	C(25)#3-C(25)-H(25A)	108.8
C(21A)-C(22A)-H(22F)	109.5	N(9)-C(25)-H(25B)	108.8
H(22D)-C(22A)-H(22F)	109.5	C(25)#3-C(25)-H(25B)	108.8
H(22E)-C(22A)-H(22F)	109.5	H(25A)-C(25)-H(25B)	107.7
C(23)-O(12)-P(4)	135.2(10)	N(12)-C(26)-N(10)	124.7(6)
C(23A)-O(12)-P(4)	115.5(9)	N(12)-C(26)-N(9)	117.3(5)
O(12)-C(23)-C(24)	107.4(17)	N(10)-C(26)-N(9)	118.0(5)
O(12)-C(23)-H(23A)	110.2	N(11)-C(27)-N(10)	127.5(5)
C(24)-C(23)-H(23A)	110.2	N(11)-C(27)-P(5)	116.4(4)
O(12)-C(23)-H(23B)	110.2	N(10)-C(27)-P(5)	116.0(4)
C(24)-C(23)-H(23B)	110.2	N(12)-C(28)-N(11)	127.0(5)
H(23A)-C(23)-H(23B)	108.5	N(12)-C(28)-P(6)	116.9(4)
C(24A)-C(23A)-O(12)	112.9(19)	N(11)-C(28)-P(6)	116.0(4)
C(24A)-C(23A)-H(23C)	109.0	O(14)-C(29)-C(30)	106.0(14)
O(12)-C(23A)-H(23C)	109.0	O(14)-C(29)-H(29A)	110.5
C(24A)-C(23A)-H(23D)	109.0	C(30)-C(29)-H(29A)	110.5
O(12)-C(23A)-H(23D)	109.0	O(14)-C(29)-H(29B)	110.5
H(23C)-C(23A)-H(23D)	107.8	C(30)-C(29)-H(29B)	110.5
C(23)-C(24)-H(24A)	109.5	H(29A)-C(29)-H(29B)	108.7
C(23)-C(24)-H(24B)	109.5	C(30A)-C(29A)-O(14)	116.5(14)
H(24A)-C(24)-H(24B)	109.5	C(30A)-C(29A)-H(29C)	108.2
C(23)-C(24)-H(24C)	109.5	O(14)-C(29A)-H(29C)	108.2
C(30A)-C(29A)-H(29D)	108.2	H(33A)-C(33)-H(33B)	107.3
O(14)-C(29A)-H(29D)	108.2	C(34A)-C(33A)-O(17)	123(2)
H(29C)-C(29A)-H(29D)	107.3	C(34A)-C(33A)-H(33C)	106.6
C(29)-C(30)-H(30A)	109.5	O(17)-C(33A)-H(33C)	106.6

C(29)-C(30)-H(30B)	109.5	C(34A)-C(33A)-H(33D)	106.6
H(30A)-C(30)-H(30B)	109.5	O(17)-C(33A)-H(33D)	106.6
C(29)-C(30)-H(30C)	109.5	H(33C)-C(33A)-H(33D)	106.6
H(30A)-C(30)-H(30C)	109.5	C(33)-C(34)-H(34A)	109.5
H(30B)-C(30)-H(30C)	109.5	C(33)-C(34)-H(34B)	109.5
C(29A)-C(30A)-H(30D)	109.5	H(34A)-C(34)-H(34B)	109.5
C(29A)-C(30A)-H(30E)	109.5	C(33)-C(34)-H(34C)	109.5
H(30D)-C(30A)-H(30E)	109.5	H(34A)-C(34)-H(34C)	109.5
C(29A)-C(30A)-H(30F)	109.5	H(34B)-C(34)-H(34C)	109.5
H(30D)-C(30A)-H(30F)	109.5	C(33A)-C(34A)-H(34D)	109.5
H(30E)-C(30A)-H(30F)	109.5	C(33A)-C(34A)-H(34E)	109.5
C(32)-C(31)-O(15)	116.5(7)	H(34D)-C(34A)-H(34E)	109.5
C(32)-C(31)-H(31B)	108.2	C(33A)-C(34A)-H(34F)	109.5
O(15)-C(31)-H(31B)	108.2	H(34D)-C(34A)-H(34F)	109.5
C(32)-C(31)-H(31A)	108.2	H(34E)-C(34A)-H(34F)	109.5
O(15)-C(31)-H(31A)	108.2	O(18)-C(35)-C(36)	115.0(8)
H(31B)-C(31)-H(31A)	107.3	O(18)-C(35)-H(35A)	108.5
C(31)-C(32)-H(32A)	109.5	C(36)-C(35)-H(35A)	108.5
C(31)-C(32)-H(32B)	109.5	O(18)-C(35)-H(35B)	108.5
H(32A)-C(32)-H(32B)	109.5	C(36)-C(35)-H(35B)	108.5
C(31)-C(32)-H(32C)	109.5	H(35A)-C(35)-H(35B)	107.5
H(32A)-C(32)-H(32C)	109.5	C(35)-C(36)-H(36A)	109.5
H(32B)-C(32)-H(32C)	109.5	C(35)-C(36)-H(36B)	109.5
O(17)-C(33)-C(34)	116.6(18)	H(36A)-C(36)-H(36B)	109.5
O(17)-C(33)-H(33A)	108.1	C(35)-C(36)-H(36C)	109.5
C(34)-C(33)-H(33A)	108.1	H(36A)-C(36)-H(36C)	109.5
O(17)-C(33)-H(33B)	108.1	H(36B)-C(36)-H(36C)	109.5
C(34)-C(33)-H(33B)	108.1	C(2)-N(1)-C(1)	124.3(5)
C(2)-N(1)-H(1N)	117.8	O(1)-P(1)-O(2)	116.8(4)
C(1)-N(1)-H(1N)	117.8	O(3)-P(1)-O(2)	99.5(4)
C(3)-N(2)-C(2)	113.9(5)	O(1)-P(1)-C(3)	109.5(3)
C(4)- N(3)-C(3)	113.4(5)	O(3)-P(1)-C(3)	107.5(3)
C(4)-N(4)-C(2)	114.3(5)	O(2)-P(1)-C(3)	105.4(3)

C(14)-N(5)-C(13)	124.6(6)	O(4)-P(2)-O(5)	116.8(4)
C(14)-N(5)-H(5N)	117.7	O(4)-P(2)-O(6)	117.0(3)
C(13)-N(5)-H(5N)	117.7	O(5)-P(2)-O(6)	100.7(3)
C(15)-N(6)-C(14)	113.4(5)	O(4)-P(2)-C(4)	111.1(3)
C(15)-N(7)-C(16)	113.2(5)	O(5)-P(2)-C(4)	105.9(3)
C(16)-N(8)-C(14)	114.6(5)	O(6)-P(2)-C(4)	104.0(3)
C(26)-N(9)-C(25)	125.1(5)	O(7)-P(3)-O(9)	116.1(3)
C(26)-N(9)-H(9N)	117.4	O(7)-P(3)-O(8)	118.8(4)
C(25)-N(9)-H(9N)	117.4	O(9)-P(3)-O(8)	99.6(3)
C(27)-N(10)-C(26)	113.6(5)	O(7)-P(3)-C(15)	110.6(3)
C(27)-N(11)-C(28)	112.7(5)	O(9)-P(3)-C(15)	105.6(3)
C(28)-N(12)-C(26)	114.5(5)	O(8)-P(3)-C(15)	104.6(3)
C(9)-O(5)-P(2)	135.4(11)	O(10)-P(4)-O(11)	118.3(4)
C(9A)-O(5)-P(2)	114.5(11)	O(10)-P(4)-O(12)	116.1(4)
C(11)-O(6)-P(2)	127.7(10)	O(11)-P(4)-O(12)	99.1(4)
C(11A)-O(6)-P(2)	123.6(8)	O(10)-P(4)-C(16)	111.2(3)
C(17)-O(8)-P(3)	128.1(10)	O(11)-P(4)-C(16)	103.7(3)
C(17A)-O(8)-P(3)	124.1(9)	O(12)-P(4)-C(16)	106.9(3)
C(21)-O(11)-P(4)	123.0(11)	O(16)-P(6)-O(17)	117.5(4)
C(21A)-O(11)-P(4)	126.0(9)	O(16)-P(6)-O(18)	116.8(4)
C(29)-O(14)-P(5)	126.3(9)	O(17)-P(6)-O(18)	98.4(4)
C(29A)-O(14)-P(5)	123.6(9)	O(16)-P(6)-C(28)	110.4(3)
C(31)-O(15)-P(5)	125.6(6)	O(17)-P(6)-C(28)	105.4(3)
C(33)-O(17)-P(6)	123.3(10)	O(18)-P(6)-C(28)	107.1(3)
C(33A)-O(17)-P(6)	126.5(11)	O(13)-P(5)-O(14)	117.4(4)
C(35)-O(18)-P(6)	126.2(7)	O(13)-P(5)-O(15)	117.4(3)
O(1)-P(1)-O(3)	117.1(4)	O(14)-P(5)-O(15)	99.4(3)
O(13)-P(5)-C(27)	110.0(3)		
O(14)-P(5)-C(27)	105.1(3)		
O(15)-P(5)-C(27)	106.2(3)		

Symmetry transformations used to generate equivalent atoms:
#1 -x-1,-y+2,-z+2   #2 -x,-y+2,-z+1   #3 -x-2,-y+1,-z

Table S9. Fractional atomic coordinates (Å×10^4) and equivalent isotropic displacement parameters (Å^2×10^3) of the non-hydrogen atoms for Pip-bis-TEPT. Ueq is defined as one third of the trace of the orthogonalised U_{IJ} tensor.

Atom	x	y	z	U(eq)
P1	3477.2(12)	7344.5(7)	11437.0(7)	42.0(3)
P2	1641.7(12)	6528.4(8)	6810.4(7)	42.1(3)
O1	1510(3)	7351(2)	11455(2)	55.6(6)
O2	5258(3)	8178(2)	12592.7(19)	50.5(6)
O3	3873(4)	6075(2)	11238(3)	66.3(7)
O4	-254(3)	6732(3)	6673(2)	60.2(7)
O5	1663(4)	5166(2)	6605(2)	52.4(6)
O6	2740(3)	6911(2)	5966.4(19)	47.9(6)
N1	2802(3)	7120(2)	9164(2)	39.6(6)
N2	5035(3)	8319(2)	8462(2)	36.1(5)
N3	5810(3)	8716(2)	10535(2)	36.5(5)
N4	7979(3)	9815(2)	9819(2)	38.6(6)
C1	4112(4)	7802(3)	10255(2)	36.4(6)
C2	3374(4)	7445(3)	8312(2)	36.5(6)
C3	6239(4)	8936(2)	9599(2)	34.6(6)
C4	9420(4)	10489(3)	11015(3)	41.6(7)
C5	8802(4)	9942(3)	8878(3)	42.6(7)
C6	5488(6)	9470(3)	13210(3)	60.5(9)
C7	7489(8)	10085(4)	14101(4)	92.2(16)
C8	2498(8)	4894(4)	10627(6)	100.4(13)
C9	2821(8)	3953(4)	11016(6)	100.4(13)
C10	481(6)	4289(3)	7004(3)	58.2(9)
C11	937(7)	3120(4)	6664(4)	70.9(11)
C12	4357(6)	6491(4)	5747(4)	61.2(9)

Table S10. Hydrogen atom coordinates (Å×10^4) and Isotropic displacement parameters (Å^{2}x10^3) for Pip-bis-TEPT.

Atom	x	y	z	U(eq)
H4A	8804.12	10332.52	11591.1	50
H4B	9841.26	11384.67	11178	50
H5A	9194.01	10812.28	8941.03	51
H5B	7800.03	9440.43	8110.75	51
H6A	4488.34	9504.54	13594.41	73
H6B	5327.38	9897.53	12654.71	73
H7A	8468.95	10052.5	13712.9	111
H7B	7634.06	9657.64	14648.21	111
H7C	7666.74	10944.89	14521.92	111
H8A	2432.67	4656.49	9804.37	121
H8B	1209.24	4934.73	10656.56	121
H9A	2721.46	4116.88	11795.36	121
H9B	4122.65	3928.83	11036.35	121
H9C	1845.02	3159.79	10484.5	121
H10A	-921.22	4122.99	6638.91	70
H10B	801.89	4642.14	7856.72	70
H11A	2310.86	3285.47	7065.19	85
H11B	676.29	2800.33	5823.72	85
H11C	122.74	2510.49	6880.2	85
H12A	3835.3	5629.31	5192.29	73
H12B	5229.19	6526.8	6479.42	73
H13A	4604.97	7292.87	4530.36	99
H13B	6538.21	7042.43	5081.88	99
H13C	6027.14	8169.31	5811.22	99

Table S11. Anisotropic displacement parameters (Å^{2}x10^3) for Pip-bis-TEPT. The Anisotropic displacement factor exponent takes the form: $-2\pi^2[h^2a^{*2}U_{11}+2hka^*b^*U_{12}+\ldots]$.

Atom	U_{11}	U_{22}	U_{33}	U23	U_{13}	U_{12}
P1	44.0(5)	40.1(4)	42.4(5)	19.0(3)	15.1(4)	9.5(3)
P2	35.8(4)	49.8(5)	36.9(4)	12.2(3)	10.9(3)	13.1(3)
O1	45.2(13)	71.4(16)	60.9(15)	33.5(12)	28.2(11)	16.7(11)
O2	57.1(14)	48.3(12)	42.9(12)	20.3(10)	9.7(10)	14.1(10)
O3	71.9(18)	44.2(13)	85.9(19)	30.4(13)	25.2(14)	17.5(12)
O4	36.6(12)	86.5(18)	48.9(13)	14.1(12)	7.1(10)	25.3(12)
O5	60.4(15)	45.2(12)	49.5(13)	13.2(10)	26.2(11)	11.3(10)
O6	50.7(13)	61.4(13)	43.5(12)	24.8(10)	21.5(10)	26.2(11)
N1	33.3(13)	42.9(13)	38.1(13)	13.6(10)	11.4(10)	6.9(10)
N2	30.6(12)	39.4(12)	41.3(13)	17.4(10)	12.9(10)	12.9(10)
N3	31.6(12)	37.5(12)	40.0(13)	14.7(10)	11.9(10)	10.3(10)
N4	28.6(12)	43.0(13)	44.7(14)	17.3(11)	14.3(10)	9.2(10)
C1	36.4(15)	35.0(13)	39.1(15)	13.3(11)	13.4(12)	13.7(12)
C2	33.0(14)	39.7(14)	38.9(15)	15.0(12)	12.2(11)	14.6(12)
C3	31.5(14)	34.6(13)	40.6(15)	14.1(11)	12.6(11)	14.9(11)
C4	33.9(15)	42.9(15)	40.5(16)	9.7(12)	13.9(12)	5.3(12)
C5	34.7(15)	49.5(16)	44.4(16)	24.3(13)	12.5(12)	7.9(13)
C6	69(2)	54(2)	52(2)	15.9(16)	15.9(17)	17.1(17)
C7	88(3)	67(3)	81(3)	20(2)	-12(3)	4(2)
C8	89(3)	58.2(18)	147(4)	43(2)	22(2)	21.4(17)
C9	89(3)	58.2(18)	147(4)	43(2)	22(2)	21.4(17)
C10	59(2)	54.9(19)	55(2)	17.8(16)	26.5(17)	5.0(16)
C11	81(3)	59(2)	81(3)	32(2)	40(2)	18(2)
C12	71(2)	77(2)	63(2)	38(2)	40.0(19)	38(2)
C13	74(3)	111(4)	88(3)	57(3)	47(2)	32(3)

Table S12. Bond angles (°) for Pip-bis-TEPT.

O1-P1-O2	117.16(14)	C3-N4-C4	122.2(2)
O1-P1-O3	117.74(15)	C3-N4-C5	122.3(2)
O1-P1-C1	112.34(13)	C4-N4-C5	113.8(2)
O2-P1-C1	106.05(13)	N1-C1-P1	114.9(2)
O3-P1-O2	98.71(14)	N3-C1-P1	118.3(2)
O3-P1-C1	102.97(14)	N3-C1-N1	126.7(3)
O4-P2-O5	118.10(15)	N1-C2-P2	114.4(2)
O4-P2-O6	114.55(14)	N2-C2-P2	119.0(2)
O4-P2-C2	111.09(14)	N2-C2-N1	126.6(3)
O5-P2-O6	102.34(12)	N2-C3-N3	124.0(3)
O5-P2-C2	103.88(13)	N4-C3-N2	118.4(3)
O6-P2-C2	105.57(13)	N4-C3-N3	117.6(2)
C6-O2-P1	120.9(2)	N4-C4-C5 #1	110.0(2)
C8-O3-P1	126.6(3)	N4-C5-C4 #1	109.7(2)
C10-O5-P2	124.2(2)	O2-C6-C7	108.4(3)
C12-O6-P2	122.5(2)	O3-C8-C9	116.6(5)
C1-N1-C2	113.0(2)	O5-C10-C11	107.9(3)
C2-N2-C3	114.9(2)	O6-C12-C13	108.7(3)
C1-N3-C3	114.7(2)		

#1 2-X,2-Y,2-Z
